# Supplementary material for: A Ce-CuZn catalyst with abundant Cu/Zn-OV-Ce active sites for CO2 hydrogenation to methanol
Source: Nat Commun. 2024 Mar 9;15:2159. doi: 10.1038/s41467-024-46513-3 (PMC10924954; doi:10.1038/s41467-024-46513-3)
Supplement: Supplementary file 1 — Supplementary Information [file 41467_2024_46513_MOESM1_ESM.pdf]

Supplementary Information

A Ce-CuZn catalyst with abundant Cu/Zn-O_V-Ce active sites for CO_2 hydrogenation to methanol

Runping Ye,[1#] Lixuan Ma,[2,#] Jianing Mao,[3,4#] Xinyao Wang,[5] Xiaoling Hong,[5] Alessandro Gallo,[6] Yanfu Ma,[5] Wenhao Luo,[7] Baojun Wang,[2] Riguang Zhang,[2,*] Melis Seher Duyar,[8,9,*] Zheng Jiang,[10,*] and Jian Liu[5,7,8*]

[1] Key Laboratory of Jiangxi Province for Environment and Energy Catalysis, Institute of Applied Chemistry, School of Chemistry and Chemical Engineering, Nanchang University, Nanchang 330031, P.R. China

[2] State Key Laboratory of Clean and Efficient Coal Utilization, College of Chemical Engineering and Technology, Taiyuan University of Technology, Taiyuan 030024, Shanxi, P.R. China

[3] Shanghai Institute of Applied Physics, Chinese Academy of Sciences, Shanghai 201204, China

[4] Center of Materials Science and Optoelectronics Engineering, University of Chinese Academy of Sciences, Beijing 100049, P.R. China

[5] State Key Laboratory of Catalysis, Dalian Institute of Chemical Physics, Chinese Academy of Sciences, Dalian 116023, Liaoning, P.R. China

[6] SUNCAT Center for Interface Science and Catalysis, SLAC National Accelerator Laboratory, 2575 Sand Hill Road, Menlo Park, California, 94025, USA

[7] College of Chemistry and Chemical Engineering, Inner Mongolia University, Hohhot 010021, P.R. China

[8] DICP-Surrey Joint Centre for Future Materials, and Advanced Technology Institute, University of Surrey, Guilford, Surrey GU2 7XH, United Kingdom

[9] School of Chemistry and Chemical Engineering, University of Surrey, Guildford, Surrey GU2 7XH, United Kingdom

[10] National Synchrotron Radiation Laboratory, University of Science and Technology of China, Hefei 230029, P.R. China

[#] These authors contributed equally to this work and should be considered co-first authors.

*Corresponding Authors

Prof. Dr. R. Zhang, Email: zhangriguang@tyut.edu.cn, ORCID: 0000-0001-8956-8425

Dr. M. S. Duyar, Email: m.duyar@surrey.ac.uk, ORCID: 0000-0002-9891-5932

Prof. Dr. Zheng Jiang, Email: jiangz@ustc.edu.cn, ORCID: 0000-0003-4297-464X

Prof. Dr. J. Liu, Email: jian.liu@surrey.ac.uk, ORCID: 0000-0002-5114-0404

Inventory of Supplementary Information

S1. Supplementary Catalyst Preparation 4

S2. Supplementary Catalyst Evaluation 6

S3. Supplementary Catalyst Characterizations 7

S4. Supplementary Computational Details 11

S4.1. Calculation Methods 11

S4.2. Surface Models 12

S5. Supplementary Tables 13

Supplementary Table 1. Surface Cu and Zn species of the reduced CuZnCe catalysts determined by quasi in-situ XPS. 13

Supplementary Table 2. Cu K-edge EXAFS curves fitting parameters. 13

Supplementary Table 3. Zn K-edge EXAFS curves fitting parameters. 14

Supplementary Table 4. Catalytic performance of the CuZnCe catalysts. 15

Supplementary Table 5. The detailed information about the TOF values over CuZnCe catalysts. 16

Supplementary Table 6. Representative catalysts for CO_2 hydrogenation to methanol. 17

Supplementary Table 7. Adsorption free energies and key structural parameters of CO_2, CO and H_2 species over O_V-CeO_2, Cu^+-CeO_{2-x} and Zn/Cu^+-CeO_{2-x} catalysts at 553 (0) K. 18

Supplementary Table 8. Calculated activation barrier (G_a), reaction energy (ΔG) of H_2 and CO_2 activation over O_V-CeO_2, Cu^+-CeO_{2-x} and Zn/Cu^+-CeO_{2-x} catalysts at 553 K. 19

S6. Supplementary Figures 20

Supplementary Fig. 1. Synthesis of CuZnCe series catalysts by different preparation routes. 20

Supplementary Fig. 2. XRD patterns and TG curves of the samples 20

Supplementary Fig. 3. The detailed XRD patterns of the dried Ce-MOF and Ce-CuZn-MOF with simulated results 21

Supplementary Fig. 4. SEM images of CuZnCe samples 21

Supplementary Fig. 5. TEM (a, b) and HRTEM (c) images of calcined Ce-CuZn sample with corresponding elemental mapping. 22

Supplementary Fig. 6. TEM images of (a) calcined and (b) reduced Ce-CuZn sample with corresponding particle size distributions. 23

Supplementary Fig. 7. SEM and TEM images: (a, b) SEM and (c, d) TEM images of CuZn-Ce sample. 23

Supplementary Fig. 8. SEM and TEM images: (a, b) SEM and (c, d) TEM images of Ce-CuZn-

IM sample. 24
Supplementary Fig. 9. Physical adsorption analysis: (a) N_2 adsorption-desorption and (b) pore size distributions of CuZnCe catalysts. 25
Supplementary Fig. 10. In-situ XRD patterns of Ce-CuZn samples reduced at different time under the atmosphere of 40%H_2-10%CO_2-N_2. 25
Supplementary Fig. 11. H_2-TPR curves of CuZnCe catalysts with corresponding fitting results. 26
Supplementary Fig. 12. Quasi in-situ XPS profiles of the reduced CuZnCe catalysts 26
Supplementary Fig. 13. Quasi in-situ XPS profiles of the reduced CuZnCe catalysts: 27
Supplementary Fig. 14. XAS spectra of reduced Ce-CuZn and Ce-CuZn-IM samples..........27
Supplementary Fig. 15. The methanol selectivity and STY of methanol under similar CO_2 conversion. 28
Supplementary Fig. 16. The Con.$_{CO2}$ and methanol selectivity as well as the STY of methanol over the Ce-CuZn catalyst *via* different reaction temperatures in CO_2 hydrogenation. 28
Supplementary Fig. 17. The Con.$_{CO}$ and product selectivity as well as the STY of methanol over the Ce-CuZn catalyst via different reaction temperatures in CO hydrogenation. 29
Supplementary Fig. 18. The result of in-situ DRIFTS for CO_2 hydrogenation (10%CO_2-40%H_2/Ar) at 260 °C and 0.1 MPa on Ce-CuZn-IM catalyst. 30
Supplementary Fig. 19. Transient in-situ DRIFTS experiments on Ce-CuZn catalyst collected at 260 °C..31
Supplementary Fig. 20. Surface morphology of samples in this work 31
Supplementary Fig. 21. The optimized structures of H_2 adsorption configurations on the models 32
Supplementary Fig. 22. The structures of initial state, transition state and final state involved in the dissociation of molecular adsorption H_2 on the models: 33
Supplementary Fig. 23. The optimized configurations of both the linear and bent CO_2 adsorption on the models: 33
Supplementary Fig. 24. The optimized configurations of CO adsorption on the models: 34
Supplementary Fig. 25. The structures of initial state, transition state and final state of CO_2 activation on the models: 35
Supplementary References...36

S1. Supplementary Catalyst Preparation

Chemicals: Cerium nitrate hexahydrate ($Ce(NO_3)_3·6H_2O$, AR), copper nitrate trihydrate ($Cu(NO_3)_2·3H_2O$, AR), zinc nitrate hexahydrate ($Zn(NO_3)_2·6H_2O$, AR), methanol (CH_3OH, AR), and ethanol (C_2H_5OH, AR) were purchased from Sinopharm Chemical Reagent Co., Ltd. 1,3,5-benzenetricarboxylic acid (1,3,5-BTC, 98%) was purchased from Aladdin. Deionized water (DI) was prepared in the laboratory. All chemicals were used without further treatment.

Synthesis of CuZnCe (Route 1): The CuZnCe was prepared by one-pot method. Namely, 0.484 g of $Cu(NO_3)_2·3H_2O$, 0.298 g of $Zn(NO_3)_2·6H_2O$ and 1.736 g of $Ce(NO_3)_3·6H_2O$ were dissolved into a solvent mixture consisting of 40 mL methanol and 40 mL ethanol (Solution A). Also, 0.840 g of 1,3,5-benzenetricarboxylate acid (1,3,5-BTC) was dissolved into a mixture solvent of 40 mL methanol and 40 mL ethanol (Solution B). Then solution B was added into solution A, with 40 mL H_2O added subsequently. After the mixture was stirred at room temperature (RT) for 20 min, it was centrifuged and washed by ethanol for several times. Finally, the mixture was dried at 60 °C f in a vacuum oven for 9 h and in a common oven for another 3 h and calcined at 450 °C for 5 h in air with a heating rate of 2 °C/min. The obtained samples were denoted as CuZnCe-MOF and CuZnCe, respectively.

Synthesis of CuZn-Ce (Route 2): The CuZn-MOF was synthesized firstly and then CuZn-Ce-MOF was prepared. Namely, 0.484 g of $Cu(NO_3)_2·3H_2O$ and 0.298 g of $Zn(NO_3)_2·6H_2O$ were dissolved into 40 mL ethanol (Solution A). Also, 0.63 g of 1,3,5-BTC was dissolved into 40 mL ethanol (Solution B). Then solution B was poured into solution A. After the mixture was stirred at RT for 60 min, it was centrifuged and washed by ethanol for several times. Finally, the mixture was dried at 60 °C in a vacuum oven for 9 h and in a common oven for another 3 h to obtain the CuZn-MOF. 0.29 g of the dried CuZn-MOF powder was dispersed into a mixture solvent of 20 mL methanol and 20 mL ethanol. Also, 1.736 g of $Ce(NO_3)_3·6H_2O$ was dispersed into a solvent mixture consisting of 20 mL methanol, 20 mL ethanol, and 20 mL H_2O. Then the cerium nitrate solution was added into the CuZn-MOF suspension under ultrasonic condition.

After sonication for 30 seconds, the mixture was stirred for 20 min at RT and then centrifuged and washed by ethanol for several times. Finally, the mixture was dried at 60 °C in a vacuum oven for 9 h and in a common oven for another 3 h and calcined at 450 °C for 5 h in air with a heating rate of 2 °C/min. The obtained samples were denoted as CuZn-Ce-MOF and CuZn-Ce, respectively. In addition, the calcination of CuZn-MOF precursor could obtain the CuZn catalyst.

Synthesis of Ce-CuZn-IM (Route 4): The Ce-CuZn-IM was synthesized similarly to the route 3 for Ce-CuZn-MOF except for the procedures after the synthesis of Ce-MOF. Namely, after synthesis of the dried Ce-MOF, it was directly calcined at 450 °C for 5 h in air with a heating rate of 2 °C/min to obtain the CeO_2 powder, which was also named as Ce catalyst. Then the CeO_2 powder (0.32 g) was impregnated with a solution of 0.484 g $Cu(NO_3)_2·3H_2O$ and 0.298 g $Zn(NO_3)_2·6H_2O$ in 10 mL H_2O. The mixture was stirred at RT for 6 h, dried at 100 °C for 12 h, and calcined at 450 °C for 5 h in air with a heating rate of 2 °C/min. The obtained sample was denoted as Ce-CuZn-IM. In addition, the synthesis of Ce-CuZn-IM-B was similar to the Ce-CuZn-IM except that the amount of $Cu(NO_3)_2·3H_2O$ and $Zn(NO_3)_2·6H_2O$ were changed to 1.310 g and 0.0305 g, respectively.

Synthesis of other control samples: The Ce-Cu and Ce-Zn samples were prepared similar to the Ce-CuZn except that $Cu(NO_3)_2·3H_2O$ or $Zn(NO_3)_2·6H_2O$ was not added in the second step. The CeCu-Zn and CeZn-Cu samples were prepared to the Ce-CuZn except that $Cu(NO_3)_2·3H_2O$ or $Zn(NO_3)_2·6H_2O$ was added in the first step with $Ce(NO_3)_3·6H_2O$. The Zn-CuCe sample could not be prepared due to Zn-MOF could not be prepared under the similar conditions. The Ce_2-$CuZn_2$ and Ce_1-$CuZn_4$ samples were also prepared similar to the Ce-CuZn except that more $Zn(NO_3)_2·6H_2O$ (0.596 g, 1.192 g) and another 1,3,5-BTC (0.848 g, 1.260 g) in 20 mL methanol were added during the second step. For the Ce-Cu-Zn and Ce-Zn-Cu samples, they were prepared by three main steps. After the Ce-Cu-MOF and Ce-Zn-MOF were prepared, they were used as precursor to exchange Zn^{2+} or Cu^{2+} instead of direct calcination, which produced Ce-Cu-Zn-MOF or Ce-Zn-Cu-MOF. Finally, they were dried and

calcined to obtain the Ce-Cu-Zn and Ce-Zn-Cu samples.

S2. Supplementary Catalyst Evaluation

The catalyst precursors (0.2 g, 20-40 meshes) mixed with 0.5 g silica sand were pre-treated at 350 °C and 1.0 MPa for 2 h under high purity hydrogen. Then the gas was changed to the reaction gases (H_2: CO_2: N_2= 72: 24: 1) with the gas hour space velocity (GHSV) set to 10,000-20,000 mL·g_{cat}^{-1}·h^{-1} under 2.0 MPa. The CO_2 conversion (Con.$_{CO2}$), methanol selectivity (Sel.), and space-time yield (STY, g·kg_{cat}^{-1}·h^{-1}) of methanol were calculated by an internal normalization method[1,2]. The products maintained at 150 °C were analyzed by on-line gas chromatography (SHIMADZU GC-2014) with flame ionization detector (FID) and thermal conductivity detector (TCD). Porapak-S 80/100 and molecular sieve-13X 80/100 packed columns were connected to TCD, whereas Rtx®-1 (60 m) and Rt®-Q-BOND (30 m) capillary columns were connected to FID. Finally, the CO_2 conversion (Con.$_{CO2}$), methanol selectivity (Sel.$_{MeOH}$), CO selectivity (Sel.$_{CO}$), and space-time yield (STY, g·kg_{cat}^{-1}·h^{-1}) of methanol were calculated using the following equations (1-5):

$$Con._{CO2} = \frac{f_{CO}A_{CO}+i(f_{CH4}A_{CH4}+f_{CH3OH}A_{CH3OH}+2f_{C2H6}A_{C2H6}+2f_{CH3OCH3}A_{CH3OCH3})}{f_{CO2}A_{CO2}+f_{CO}A_{CO}+i(f_{CH4}A_{CH4}+f_{CH3OH}A_{CH3OH}+2f_{C2H6}A_{C2H6}+2f_{CH3OCH3}A_{CH3OCH3})} \quad (1),$$

$$Sel._{CH3OH} = \frac{if_{CH3OH}A_{CH3OH}}{f_{CO}A_{CO}+i(f_{CH4}A_{CH4}+f_{CH3OH}A_{CH3OH}+2f_{C2H6}A_{C2H6}+2f_{CH3OCH3}A_{CH3OCH3})} \quad (2),$$

$$Sel._{CO} = \frac{f_{CO}A_{CO}}{f_{CO}A_{CO}+i(f_{CH4}A_{CH4}+f_{CH3OH}A_{CH3OH}+2f_{C2H6}A_{C2H6}+2f_{CH3OCH3}A_{CH3OCH3})} \quad (3),$$

$$STY_{CH3OH} = \frac{GHSV}{1000 \times 22.4} \times V\%(CO_2) \times Conv._{CO2} \times Select._{CH3OH} \times M_{CH3OH} \quad (4),$$

$i = \frac{f_{CH4-TCD}A_{CH4-TCD}}{f_{CH4-FID}A_{CH4-FID}}$ (5), where *"f"* is the relative mole correction factor, *"A"* is the peak area on chromatographic spectra, and *"i"* is the internal normalization conversion coefficient.

S3. Supplementary Catalyst Characterizations

The characterization methods were referenced to this work and depicted details as following part[3].

ICP test: The inductively coupled plasma optical emission spectroscopy (PerkinElmer ICP-OES 7300DV) was used to determine the actual Cu, Zn, and Ce contents.

Nitrogen sorption test: About 100 mg of samples were firstly degassed at 150 °C for 11 h, then it was measured at 77 K in micromeritics TriStar II 3020. The Brunauer-Emmett-Teller (BET) method was used to analyze the specific surface area (S_{BET}). The total pore volume (V_p), average pore diameter (D_p), and pore size distributions were determined by the Barrett-Joyner-Halenda (BJH) method using the adsorption isotherm.

TG test: About 10 mg of the sample was filled into a crucible and then it was heated to 800 °C with a heating rate of 5°C/min under the air atmosphere. The set-up type for thermogravimetric (TG) test is Mettler Toledo TGA/DSC 3+.

XRD test: The phase and crystal structures were determined by X-ray powder diffraction (XRD) patterns from 10 to 85 or 5 to 60 degree (2θ) on a Rigaku D/Max 2500/PC using the Cu Kα radiation with the voltage of 40 kV and current of 200 mA. The in-situ XRD tests were operated on a XRK 900 using Cu as anode material and the generators setting as 40 kV and 40 mA. The 2θ was set from 25 to 53 degree. The calcined Ce-CuZn sample was treated in the atmosphere of 40%H_2-N_2. and 40%H_2-10%CO_2-N_2, respectively.

Raman test: The Raman spectroscopy excited with a 532 nm laser was performed on NanoWizard Ultra Speed &in Via Raman.

H_2-TPR and CO_2-TPD tests: The H_2 temperature-programmed reduction and CO_2 temperature-programmed desorption were carried out in AutoChem II 2920 and AutoChem 2910 instrument, respectively. For the H_2-TPR test, about 80 mg sample was pretreated at 250 °C for 1 h under the Ar atmosphere. Then the temperature was decreased to 50 °C and the 10%H_2-Ar was introduced when the baseline was stable. The data was recorded from 50 to 450 °C with a heating rate of 10 °C/min. For the CO_2-

TPD test, about 100 mg sample was reduced at 350 °C for 2 h using the 10%H_2-Ar. The system was then decreased to 100 °C using the He and then introduced the highly pure CO_2 for 30 min. After dosing, the physically adsorbed CO_2 was removed by flowing He for another 30 min. Then the sample was heated to 800 °C with a heating rate of 10 °C/min under He flow. Exhaust gases were detected by a mass spectrometer (MS).

The metallic copper surface area (S_{Cu}) and copper dispersion (D_{Cu}) tests: They were also performed on Autochem II 2920 instrument by one N_2O titration and two H_2-TPR processes. Namely, the sample was firstly pretreated at 150 °C for 0.5 h under the 20%O_2-He flow. Then, the system was changed to Ar to purge the oxygen for 5 min. 10%H_2-Ar was used to reduce the sample at 350 °C with a cold trap and then decreased to 50 °C under the Ar atmosphere. N_2O (>99.9%) was applied to oxidize the metallic copper at 50 °C for 1 h. Finally, the sample was purged by Ar for 30 min and then reduced again at 350 °C by 10%H_2-Ar.

The oxygen vacancies tests: They were also performed on Autochem II 2920 instrument by one CO_2 adsorption, two N_2O pulsing titrations and two H_2-TPR processes in one sample test. The CO_2 adsorption was used to block oxygen vacancies in CeO_2-based metal catalysts. This method was referenced from Zhu et al., who reported to determine the amount of oxygen vacancies (N_{OV}) over Cu/CeO_2 catalysts[4]. Namely, the sample (~100 mg) was firstly pretreated at 350 °C for 1 h under the 10%H_2-Ar flow followed by cooling to 50 °C in a Ar flow. Next, a 5% N_2O-He gas mixture was periodically injected into the sample using a 0.58 mL sample loop until no N_2O consumption was observed. This N_2O consumption amount is denoted as $n_{N2O,\,1}$. Before the second N_2O experiment, the same sample was purged by Ar for 0.5 h and then reduced at 350 °C for 1 h under the 10%H_2-Ar flow followed by cooling to 50 °C in a Ar flow again. After cooling to 50 °C in a Ar flow, pulses of pure CO_2 were injected to the sample to block the ceria oxygen vacancies. After the CO_2 pulsing, the same N_2O pulsing experiment was carried out to titrate the remaining metallic copper sites. This N_2O consumption amount is denoted as $n_{N2O,\,2}$. The N_2O consumption by oxygen vacancies was derived from the difference between these two N_2O titration experiments

and the ratio of N_2O to N_{OV} was set as 1. Finally, the number of oxygen vacancies (N_{OV}) was calculated by the following equation (6): $N_{OV}= \frac{n_{N2O,1}-n_{N2O,2}}{m_{cat}}$ (6).

Then, the system was changed to Ar to purge the oxygen for 5 min. 10%H_2-Ar was used to reduce the sample at 350 °C with a cold trap and then decreased to 50 °C under the Ar atmosphere. N_2O (>99.9%) was applied to oxidize the metallic copper at 50 °C for 1 h. Finally, the sample was purged by Ar for 30 min and then reduced again at 350 °C by 10%H_2-Ar.

TEM and SEM tests: The transmission electron microscopy (TEM) and high-angle annular dark-field scanning transmission electron microscopy HAADF-STEM were operated on several facilities including HITACHI HT7700, TECNAI G2 F30, JEOL JEM-F200 and JEOL JEM-ARM200F. All the catalysts were dispersed into ethanol to prepare the TEM samples. The scanning electron microscopy (SEM) was performed on JSM-7900F and HITACHI S-5500.

XAS test: X-ray absorption spectroscopy (XAS) measurements were carried out at the XAS Beamline at the Australian Synchrotron (ANSTO) in Melbourne, Australia using a set of liquid nitrogen cooled Si(311) monochromator crystals. The electron beam energy is 3.0 GeV. With the associated beamline optics (Si-coated collimating mirror and Rh-coated focusing mirror), the harmonic content of the incident X-ray beam was negligible. A Ge 100 element detector was used to collect the fluorescence signal, and the energy was calibrated using Cu/Zn foils. The beam size was about 1x1mm. Note that a single XAS scan took ~1 h. Furthermore, the Cu K-edge and Zn K-edge XAFS data were processed by using the ATHENA module implemented in the IFEFFIT software packages. First, subtracting the post-edge background from the overall absorption and normalizing with respect to the edge jump step to obtain EXAFS spectra is necessary. Then, χ(k) data in the k-space (ranging from 3.0 to 11 $Å^{-1}$ for Cu and Zn) were Fourier transformed to real (R) space using hanning windows. The theoretical scattering amplitudes, phase shifts, and the photoelectron mean free path for all paths were calculated by using a hanning window (dk = 1.0 $Å^{-1}$) in IFEFFIT package.

Quasi in-situ XPS test: Quasi in-situ X-ray photoelectron spectroscopy (XPS)

experiments were carried out on Thermo Scientific Escalab 250Xi+. Before the test, the samples were pre-reduced in an atmospheric processing chamber at 350 °C for 1 h under the 20%H_2-Ar atmosphere, which was interconnected with the ultra-high vacuum chamber of XPS analysis chamber to avoid the influence of air exposure. The set-up was equipped with a monochromatic Al Kα X-ray radiation source (hv = 1486.6 eV). The beam diameter was 500 μm, and the acceleration voltage was 15 kV.

EPR test: Electron paramagnetic resonance (EPR) measurements were performed at room temperature on a Bruker A 200 spectrometer. 10 mg of sample was filled in the capillary tube for each measurement at room temperature. The detailed operation parameters were as below: The center field was 3320.00 G, the microwave frequency was 9.3296 GHz, the modulation frequency was 100.00 kHz, and the conversion time was 82 ms. The g value was determined from precise frequency and magnetic field values.

In-situ DRIFTS test: In-situ Diffuse Reflectance Infrared Fourier Transform Spectroscopy (DRIFTS) was used to reveal the copper species and potential reaction mechanism on the Bruker VERTEX 80V. The samples were firstly pre-reduced in a tube furnace at 350 °C for 2 h under the 5%H_2-Ar atmosphere. During the test, the sample was in-situ reduced again at 350 °C for 30 min using the high-pure H_2. The CO-DRIFTS was operated at 30 °C to collect the background and 5%CO-He was introduced and kept for 20 min. Then the gas was changed to He and recoded the spectra at different purging time. The Ce-CuZn and Ce-CuZn-IM catalysts were performed for in-situ CO_2 hydrogenation. After the above pre-reduction step, the system was purged with Ar or He for 20 min at 350 °C and another 30 min at 260 °C to collect the background. The 10%CO_2-40%H_2-Ar was introduced to collect the spectra (64 scans, 4 cm^{-1}) under atmospheric pressure and different dosing time at 260 °C. For the transient in-situ DRIFTS test, the pretreatment of the Ce-CuZn sample was the same as the previous procedure. After reduction, the temperature was fixed at 260 °C. Then pure CO_2 or H_2, 5%CO_2-20%H_2-He, or pure Ar were switched and introduced to the cell to collect the spectra under atmospheric pressure and different dosing times.

S4. Supplementary Computational Details

S4.1. Calculation Methods

Spin-polarized density functional theory (DFT) calculations were carried out with the Vienna Ab Initio Simulation Package (VASP) code[5,6]. The projector augmented wave (PAW) method was adopted to treat the ion-electron interactions, and the generalized gradient approximation (GGA) with Perdew-Burke-Ernzerhof (PBE) was employed for the exchange correlation function[7]. A kinetic cutoff energy was set to 400 eV for describing the electronic wave function. A vacuum layer was set to 15 Å to eliminate slab-slab interactions. The total energies were converged to 10^{-5} eV, and the forces acting on the relaxed ions were less than 0.02 eV/Å for structure optimizations. In order to account for the strong localization of Ce *4f* and Cu, Zn *3d* electrons of CuZnCe catalyst, a Hubbard-like U term was used, (GGA+U), an effective parameter U-J=5 eV was set for Ce atom, and U-J=5 eV for Cu atom, and U-J=4.7 eV for Zn atom[8]. The climbing-image nudged elastic band (CI-NEB) method was employed to search the transition states (TS)[9,10], the dimer method was used to optimize the located TS[11,12]. Moreover, the only one imaginary frequency of TS structure is confirmed.

Given the process of methanol synthesis from CO_2 hydrogenation is carried out at the temperature range of 473-573 K, the adsorption free energy (G_{ads}), activation barrier (G_a) and reaction energy (ΔG) involving in this study were calculated at 553 K using VASPKIT code[13]. The expressions are as following equations (7-9):

$$G_{ads}(\mathrm{T, P})=E_{mol/surface}-E_{mol}-E_{surface}+\Delta U+\Delta E_{ZPE}-T\Delta S \quad (7)$$

$$G_{a}(\mathrm{T, P})=E_{TS}-E_{IS}+\Delta E_{ZPE}+\Delta U-T\Delta S \quad (8)$$

$$\Delta G(\mathrm{T, P})=E_{FS}-E_{IS}+\Delta E_{ZPE}+\Delta U-T\Delta S \quad (9)$$

Where $E_{mol/surface}$, E_{mol}, and $E_{surface}$ are the total energies of the surface with adsorbate, the gaseous adsorbate molecule and the optimized surface, respectively. E_{IS}, E_{TS} and E_{FS} are the total energies of the initial state (IS), transition state (TS) and final state (FS), respectively. ΔE_{ZPE} is the change of zero-point energy (ZPE) at 0 K. ΔU and ΔS origin from the contribution of thermal energy and entropy, respectively.

S4.2. Surface Models

$CeO_2(111)$ as the most stable surface in CeO_2 bulk was modeled by a three-layer $p(3\times3)$ supercell[14], resulting from the optimized CeO_2 bulk with the lattice parameters of $a=b=c=5.43$ Å (Supplementary Fig. 20a), which agree with the previously theoretical value of 5.42 Å and the experimental value of 5.41 Å[14,15]. The defective $CeO_2(111)$ surface was modeled by removing a O atom adjacent to Ce atom, denoted as O_V-CeO_2 (Supplementary Fig. 20b). Two neighboring Ce^{4+}-O^{2-} moiety on the $CeO_2(111)$ surface are replaced by two Cu^{2+} cations and two oxygen vacancies (Cu^{2+}-O_V). To obtain surficial Cu^+, the oxygen atom connected with two Cu atoms is removed to balance the charge, named as Cu^+-CeO_{2-x} (Supplementary Fig. 20c). Taking the effect of Zn doping into account, a Ce atom on $CeO_2(111)$ surface is replaced by a Zn atom and a O atom bonded to Zn atom are removed to keep charge balance, named as Zn/Cu^+-CeO_{2-x} (Supplementary Fig. 20d).

S5. Supplementary Tables

Supplementary Table 1. Surface Cu and Zn species of the reduced CuZnCe catalysts determined by quasi in-situ XPS.

Samples	$Cu^+/(Cu^0+Cu^+)$ (%)	$O_\beta/(O_\alpha+O_\beta+O_\gamma)$ (%)	Ce^{3+} BE (eV)	Ce^{4+} BE (eV)	$Ce^{3+}/(Ce^{3+}+Ce^{4+})$ (%)
**CuZn-Ce**	52.1	25.8	881.4, 884.8<br>898.5, 903.2	882.5, 888.8, 897.7<br>900.9, 907.4, 916.7	36.4
**Ce-CuZn**	56.5	29.9	881.4, 884.6<br>898.4, 902.9	882.3, 888.6, 897.7<br>900.8, 907.3, 916.6	34.8
**Ce-CuZn-IM**	-	21.3	881.4, 884.8<br>898.8, 902.9	882.6, 889.0, 898.1<br>901.0, 907.6, 916.8	27.4

Supplementary Table 2. Cu K-edge EXAFS curves fitting parameters.

Samples	Coordination	N	R (Å)	ΔE_0 (eV)	$\sigma^2(10^{-3}Å^2)$	R-factor
Ce-CuZn	Cu-O	0.2±0.9	1.84±0.08	4.3±0.9	5.6±0.6	0.003
	Cu-Cu	10.0±0.8	2.54±0.01	4.3±0.9	9.2±0.8	
Ce-CuZn-IM	Cu-O	0.3±1.0	1.85±0.15	4.4±1.1	10.0±5.6	0.005
	Cu-Cu	8.0±0.7	2.54±0.01	4.4±1.1	8.4±0.9	

Supplementary Table 3. Zn K-edge EXAFS curves fitting parameters.

Samples	Coordination	N	R (Å)	ΔE_0 (eV)	$\sigma^2(10^{-3}Å^2)$	R-factor
Ce-CuZn	Zn-O	4.5±1.4	1.95±0.04	-1.7±3.4	5.9±3.2	0.019
	Zn-Zn	11.5±1.4	3.23±0.02	-1.7±3.4	10.0	
Ce-CuZn-IM	Zn-O	3.3±2.1	1.98±0.05	6.8±7.6	6.8±7.3	0.019
	Zn-Zn	12.0±4.3	3.24±0.04	3.0±5.2	7.0	

Notes: N, coordination number; R, distance between absorber and backscatter atoms; σ^2, Debye Waller factor; ΔE_0, inner potential correction accounting for the difference in the inner potential between the sample and the reference compound.

Supplementary Table 4. Catalytic performance of the CuZnCe catalysts.

Catalysts	Con. $_{CO2}$ %	Sel. $_{CH3OH}$ %	STY_{CH3OH} g kg_{cat}^{-1} h^{-1}	Remark
**Ce**	0.38	0	0	The CeO_2 support without Cu or Zn has no activity.
**CuZn**	0.9	38.4	11.7	CuZn without CeO_2 support would be sintering and has poor activity.
**Ce-Cu** [b)]	9.7	16.9	58.1	The sample without Zn promoter has low performance.
**Ce-Zn** [c)]	0.17	88.2	5.5	The sample without main Cu metal has bad performance.
**CuZnCe**	1.4	15.3	7.4	The sample only grew Ce with very low CuZn and has a bad performance. The three metals could not be added together.
**CuZn-Ce**	4.6	22.1	36.0	Zn was lost during preparation and has low performance.
**Ce-CuZn**	**15.1**	**26.4**	**140.6**	**The sample has high Cu/Zn-O_V-Ce species and exhibits the best performance.**
**CeCu-Zn**	1.05	34.2	12.7	The sample with low Cu content has bad performance due to CeCu-MOF could not be grown together.
**CeZn-Cu**	5.3	20.5	38.6	The sample with low Zn content has bad performance due to CeZn-MOF could not be grown together.
**Zn-CuCe**	NA	NA	NA	The sample could not be prepared due to Zn-MOF could not be prepared under similar conditions.
**Ce_2-$CuZn_2$**	10.9	38.4	148.6	The samples with increased Zn content would decrease CO_2 conversion although the methanol yield was slightly increased.
**Ce_1-$CuZn_4$**	8.9	47.4	149.6	
**Ce-Cu-Zn** [d)]	10.7	43.3	165.7	The two samples also have good performance, but the preparation procedures are more complicated.
**Ce-Zn-Cu** [d)]	12.4	23.9	106.2	
**Ce-CuZn-IM**	1.6	46.1	26.8	The sample prepared with impregnation method (IM) has lower performance than Ce-CuZn.
**Ce-CuZn-IM-B**	3.0	42.6	56.7	The sample with similar Cu and Zn loading but still has lower performance than Ce-CuZn.

[a)] Reaction conditions: 280 °C, 2 MPa, 10,000 h^{-1}, $H_2/CO_2/N_2$=72/24/1.

[b)] "-" means two main steps for the sample preparation. The Ce-MOF was grown first and then the Cu-MOF was grown on the Ce-MOF. It is the same for other samples.

[c)] Reaction temperature for this sample was 320 °C due to its low performance.

[d)] "- -" means three main steps for the sample preparation.

Supplementary Table 5. The detailed information about the TOF values over CuZnCe catalysts.[a]

**Catalysts**	**Con.$_{CO2}$ (%)**	**Flow$_{CO2}$ (mL/min)**	**m$_{catalyst}$ (g)**	**Cu (wt.%)**	**D$_{Cu}$ (%)**	**TOF[b] (h^{-1})**
CuZn-Ce	2.32	8.256	0.2	52.33	9.2	3.4
Ce-CuZn	9.59	8.256	0.2	52.82	6.7	19.0
Ce-CuZn-IM	1.12	8.256	0.2	22.36	15.2	2.3

a) Reaction conditions: *P*= 2.0 MPa, *T*= 260 °C, *GHSV*= 10,000 mL·g_{cat}^{-1}·h^{-1}, H_2: CO_2: N_2= 72: 24: 1.

b) Turnover frequency (TOF) values were calculated using Cu active sites according to this equation (10):[16] TOF= $\frac{moles\ of\ reactive\ CO_2}{moles\ of\ active\ sites \times reaction\ time}$ (10).

Supplementary Table 6. Representative catalysts for CO_2 hydrogenation to methanol.

Catalysts	Con.[a] %	Sel.[b] %	STY[c]	*T*, ℃	*P*, MPa	*GHSV*[d]	H_2/CO_2 ratio	Ref.
Cu/SiO_2-AE	28	21.3	260	320	3	16,000	4	16
Cu/ZnO@m-SiO_2	11.9	61.8	153.9	270	5	6,000	3	17
Cu/ZnO/SiO_2	14.1	57.2	55.4	220	3	2,000	3	18
Si-Cu-Zn	8.3	19	97.4	280	3	18,000	3	19
CuZnGa/SiO_2	5.6	99.5	348.8	270	2	18,000	3	20
Cu/Ga_2O_3/ZrO_2	13.7	75.6	61.76	250	2	2,500	3	10
Cu/Zn/Al/Y	26.9	52.4	520	250	5	12,000	3	21
CuZnAl-C-1.25	16.2	63.8	130	240	3	3,600	3	22
Cu/ZnO/Al_2O_3-1	23.1	31.2/NA[e]	123.7[g]	260	3	6,000	3	23
Cu/ZnO/Al_2O_3-2	18.2	41.1/NA[e]	410	260	5	15,000	3	24
Cu/ZnO/Al_2O_3-3	19.7	39.7/59.7[e]	340	250	5	12,000	3	21
Cu/ZnO/Al_2O_3-4	11.1	54.8/45.2[e]	391.3[g]	250	4	18,000	3	25
Cu/ZnO/Al_2O_3-5	13.4	58.1/41.6[e]	250	230	5	10,000	3	26
Cu/ZnO/Al_2O_3-6	24.6	67.1/32.9[e]	283.2[g]	260	5	5,000	3	27
$Cu_1La_{0.2}$/SBA-15	6	81.2	200.7[g]	240	3	12,000	3	28
Cu/ZnO/ZrO_2	15.7	58.0	117.2	240	3	3,600	3	29
ZrO_2/Cu	~4.4[f]	~70[f]	524	220	3	48,000	3	30
Cu/CeO_2	10.0	~75[f]	268[g]	280	3	10,000	3	31
Cu/TiO_{2-x}-500	~13[f]	~50[f]	139.4	300	4	6,000	3	32
Cu/ZnO-CeO_2	~6.0	~45[f]	463.2[g]	250	3	60,000	3	33
Cu-Pd/CeO_2	16.1	26.7	40.6[g]	260	3	3,000	3	34
Cu-Ni/CeO_2-NR	18.4	73.3	231.4[g]	260	3	6,000	3	35
Cu/Al_2O_3-CeO_2	~17[f]	~45[f]	364.0[g]	260	3	14,400	3	36
CuZn@UiO-bpy	3.3	100	212.3[g]	250	4	18,000	3	25
CuZn@MOF-253	8.7	93.2	46.4[g]	250	4	1,600	3	25
DSM-CuZn@UiO-67	8.9	70.8	36.0[g]	250	4	1,600	3	25
CuZn-BTC	20.9	58.2	62.6[g]	250	4	1,500	3	37
In_2O_3	7.1	39.7	118.1	330	4	15,000	3	38
h-In_2O_3-R	5.1	95.1	332.8	300	5	20,000	3	39
In_2O_3@Co_3O_4(ZIF-67)	12.5	83	510[g]	270	5	17,200	4	40
Pd-ZnO@ZIF-8	9.3	74	470	270	4.5	19,200	3	41
Ni_5Ga_3/ZrO_2	5	27	30.9	270	3.0	8,000	3	42
**Ce-CuZn-MOF**	**8.0**	**71.1/26.7[e]**	**400.3**	**260**	**2.8**	**20,000**	**3**	**h**

[a] CO_2 conversion. [b] Selectivity of methanol. [c] Space time yield of methanol ($g\cdot kg_{cat}\cdot h^{-1}$). [d] Gas hourly

space velocity ($mL\cdot g_{cat}^{-1}\cdot h^{-1}$). [e] The former data is the selectivity of methanol while the latter one is the selectivity of CO, and NA is the abbreviation of not available. [f] The data was read from the figure in the original references. [g] Calculated according to the data. [h] This work.

Supplementary Table 7. Adsorption free energies and key structural parameters of CO_2, CO and H_2 species over O_V-CeO_2, Cu^+-CeO_{2-x} and Zn/Cu^+-CeO_{2-x} catalysts at 553 (0) K.

Catalysts	Species	G_{ads} (kJ·mol^{-1})	Adsorption site	d_{C-O}/d_{H-H} (Å)
O_V-CeO_2	CO_2 (linear)	-14.3 (-79.9)	O_V	1.167,1.186
	CO_2 (bent)	-30.6 (-105.8)	Ce-O bridge	1.269,1.268
	CO (mol)	37.7 (-36.7)	Ce-top	1.141
	H_2 (mol)	-19.3 (-70.1)	Ce-top	0.758
Cu^+-CeO_{2-x}	CO_2 (linear)	55.9 (-19.1)	O_V	1.170,1.181
	CO_2 (bent)	-48.2 (-124.1)	Ce-O bridge	1.289,1.264
	CO (mol)	23.8 (-61.3)	Cu-top	1.167
	H_2 (mol)	23.0 (-5.5)	Cu-Cu bridge	0.754
Zn/Cu^+-CeO_{2-x}	CO_2 (linear)	27.1 (-43.9)	O_V	1.172,1.181
	CO_2 (bent)	-58.5 (-140.4)	Ce-O bridge	1.252,1.316
	CO (mol)	18.3 (-87.5)	Cu-Cu bridge	1.171
	H_2 (mol)	1.7 (-7.4)	Cu-Cu bridge	0.754

[*]Parentheses: adsorption energies at 0 K.

Supplementary Table 8. Calculated activation barrier (G_a), reaction energy (ΔG) of H_2 and CO_2 activation over O_V-CeO_2, Cu^+-CeO_{2-x} and Zn/Cu^+-CeO_{2-x} catalysts at 553 K.

Catalysts	Reactions	G_a (kJ·mol^{-1})	ΔG (kJ·mol^{-1})
O_V-CeO_2	H_2→H*+H*	133.1	-143.3
	CO_2+H*→HCOO*	324.8	1.6
	CO_2+H*→COOH*	364.2	64.6
Cu^+-CeO_{2-x}	H_2→H*+H*	109.1	-229.1
	CO_2+H*→HCOO*	164.8	155.2
	CO_2+H*→COOH*	225.0	217.9
Zn/Cu^+-CeO_{2-x}	H_2→H*+H*	84.8	-230.9
	CO_2+H*→HCOO*	56.0	-35.2
	CO_2+H*→COOH*	148.7	84.3
	HCOO*+H*→HCOOH*	131.2	-60.1
	HCOO*+H*→H_2COO*	63.1	44.1
	HCOOH*+H*→H_2COOH*	-	-22.4
	H_2COO*+H*→H_2COOH*	-	-1.4
	H_2COOH*→H_2CO*+OH*	-	-18.5
	H_2CO*+H*→CH_3O*	-	-127.3
	CH_3O*+H*→CH_3OH*	-	-118.0

S6. Supplementary Figures

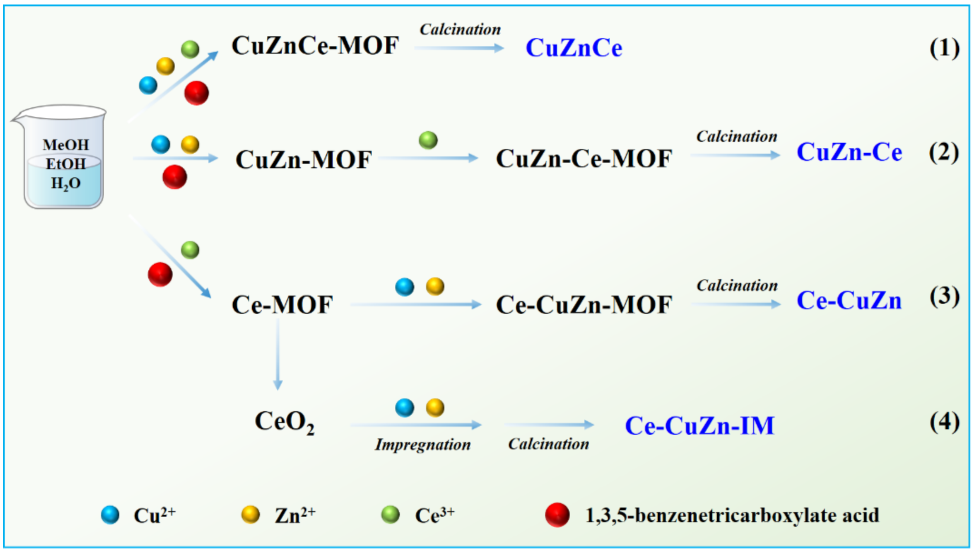

Supplementary Fig. 1. Synthesis of CuZnCe series catalysts by different preparation routes. Four routes are depicted in the figure to synthesize CuZnCe catalysts with different steps.

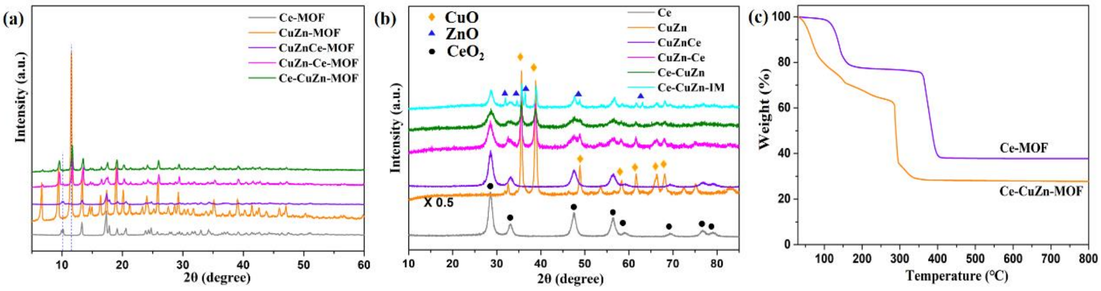

Supplementary Fig. 2. XRD patterns and TG curves of the samples: (a) Dried and (b) calcined CuZnCe catalysts. (c) TG curves under the air atmosphere.

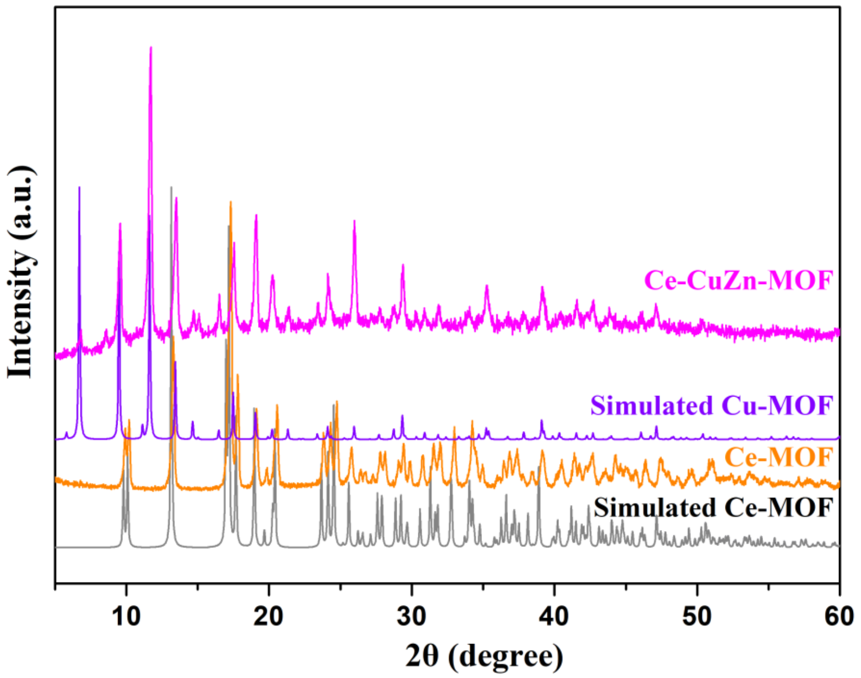

Supplementary Fig. 3. The detailed XRD patterns of the dried Ce-MOF and Ce-CuZn-MOF with simulated results. The XRD diffraction peaks of Ce-MOF are similar to the simulated Ce-MOF while the XRD patterns over Ce-CuZn-MOF changed upon the introduction of Cu^{2+}/Zn^{2+} into Ce-MOF.

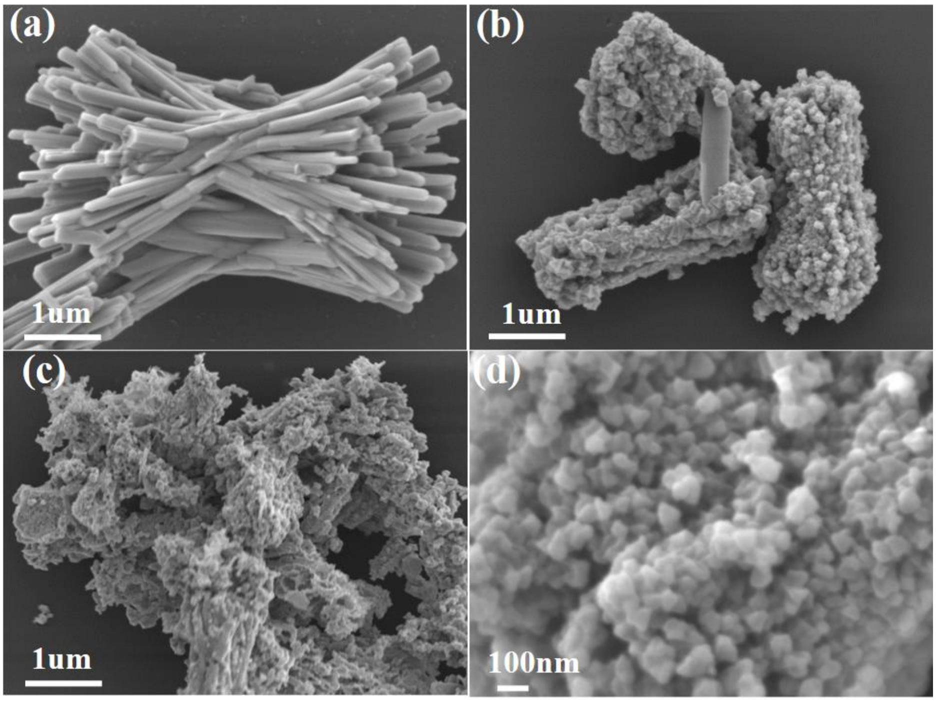

Supplementary Fig. 4. SEM images of CuZnCe samples: (a) dried Ce-MOF, (b, d) dried Ce-CuZn-MOF, (c) reduced Ce-CuZn.

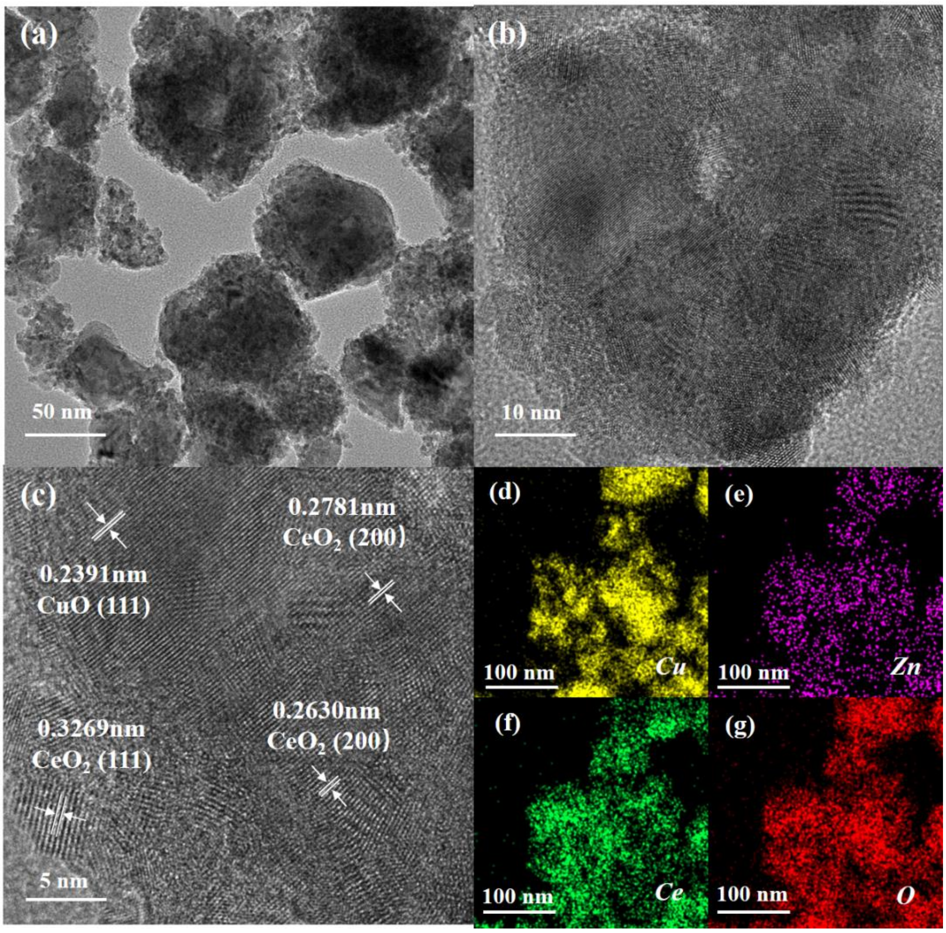

Supplementary Fig. 5. TEM (a, b) and HRTEM (c) images of calcined Ce-CuZn sample with corresponding elemental mapping (d-g). It indicates that Cu NPs are highly dispersed in the catalyst precursors.

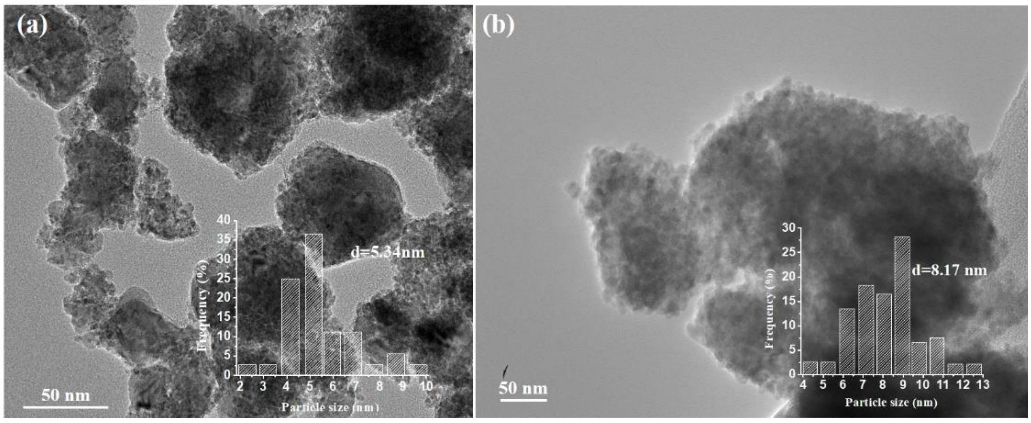

Supplementary Fig. 6. TEM images of (a) calcined and (b) reduced Ce-CuZn sample with corresponding particle size distributions. The mean Cu nanoparticle sizes were increased from 5.34 nm over the calcined Ce-CuZn to 8.17 nm over the reduced Ce-CuZn, indicating that the Cu nanoparticles were accompanied by slight migratory agglomeration during reduction process.

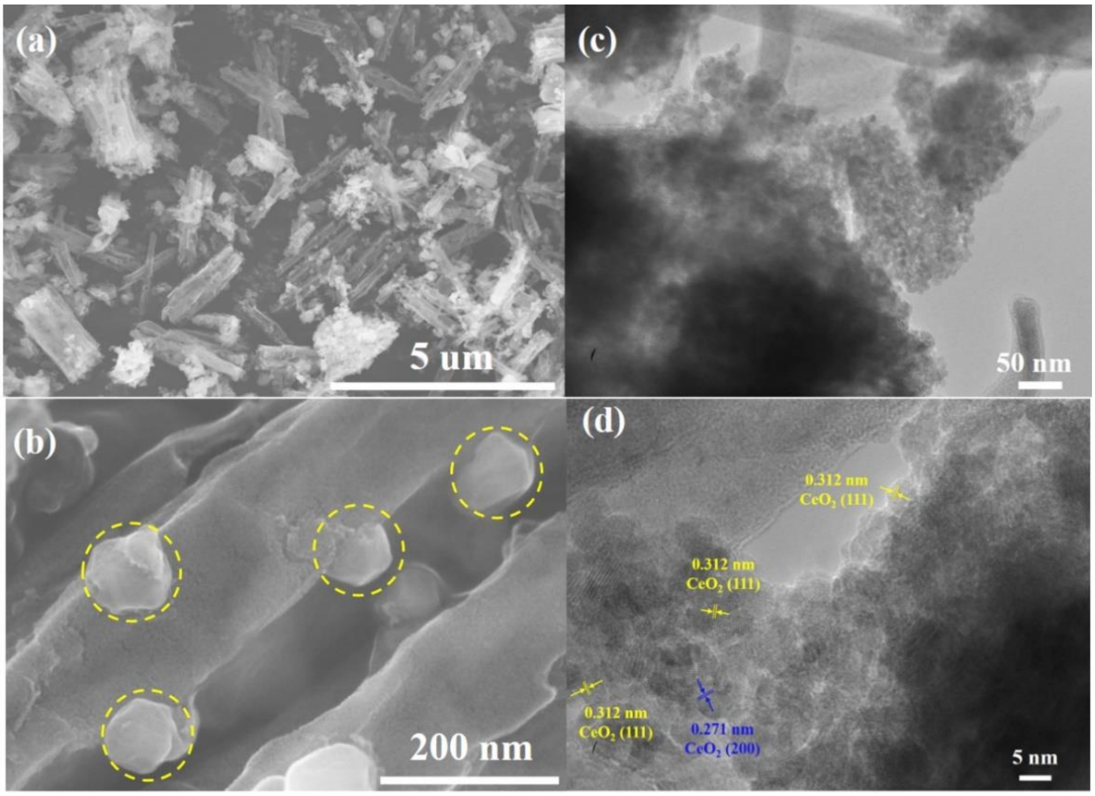

Supplementary Fig. 7. SEM and TEM images: (a, b) SEM and (c, d) TEM images of CuZn-Ce sample. The nanorods combined with the nanoparticles could also be observed over CuZn-Ce sample.

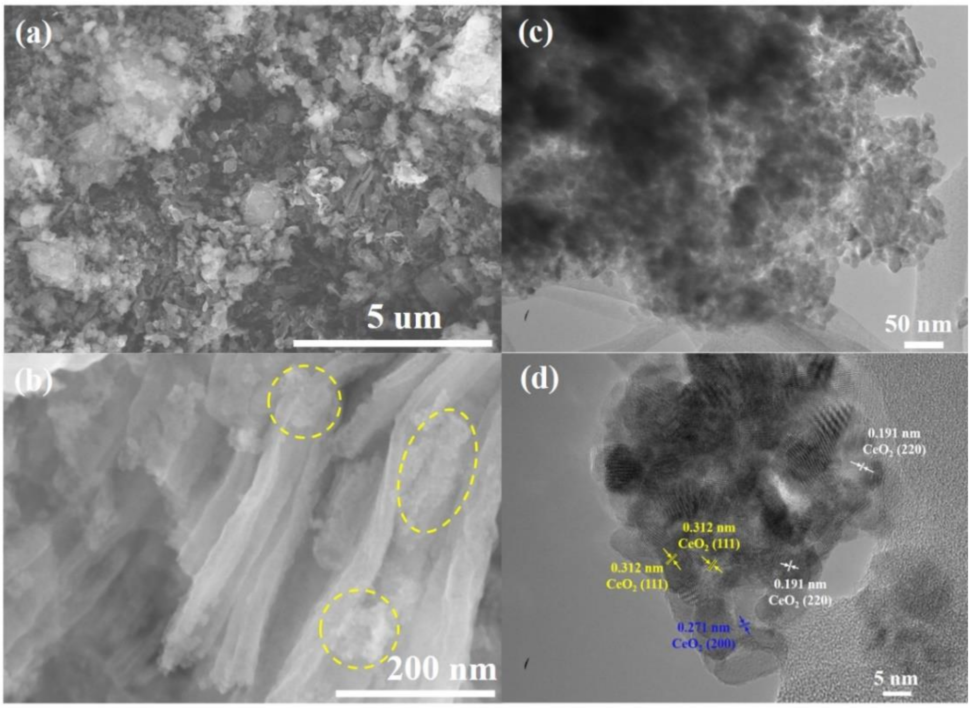

Supplementary Fig. 8. SEM and TEM images: (a, b) SEM and (c, d) TEM images of Ce-CuZn-IM sample. The nanorods combined with the nanoparticles could also be observed over Ce-CuZn-IM sample.

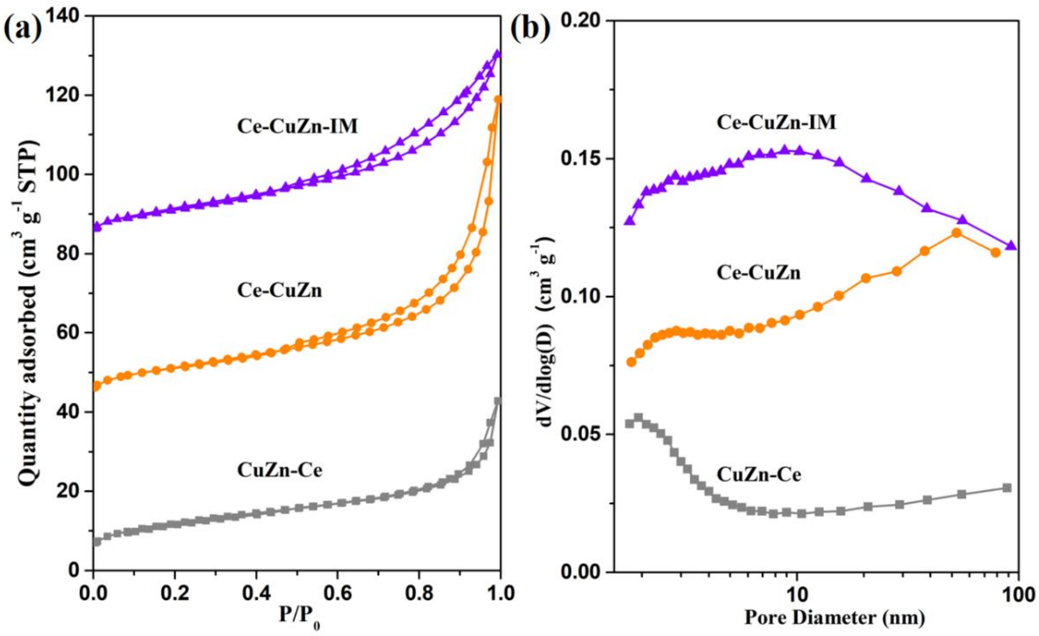

Supplementary Fig. 9. Physical adsorption analysis: (a) N_2 adsorption-desorption and (b) pore size distributions of CuZnCe catalysts. This figure shows that CuZnCe samples present a similar hysteresis loop and specific surface area, but different pore distributions.

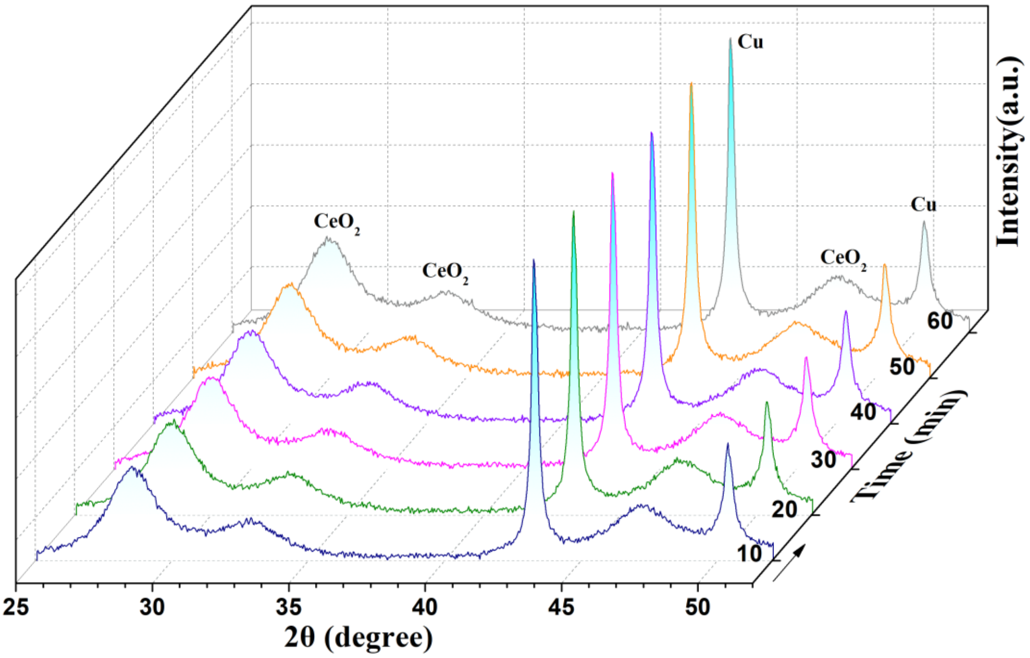

Supplementary Fig. 10. In-situ XRD patterns of Ce-CuZn samples reduced at different time under the atmosphere of 40%H_2-10%CO_2-N_2. Other reaction conditions: P= 0.1 MPa, T= 260 °C.

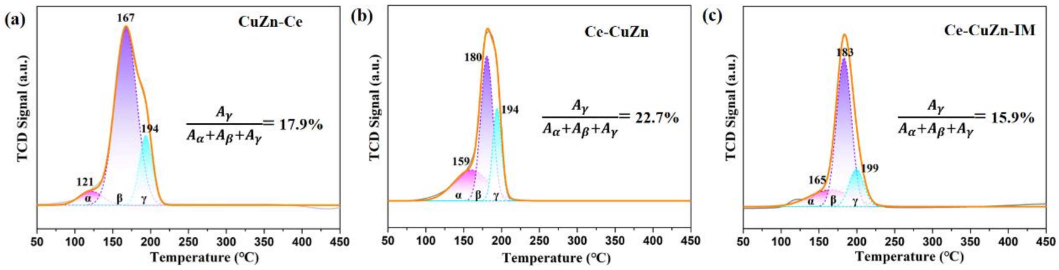

Supplementary Fig. 11. H_2-TPR curves of CuZnCe catalysts with corresponding fitting results: (a) CuZn-Ce, (b) Ce-CuZn, (c) Ce-CuZn-IM. The original curves were fitted with three peaks, which were attributed to the reduction of dispersed copper species that weakly interact with CeO_2 (peak α), bulk CuO and dispersed copper species that strongly interact with CeO_2 (peak β), and Cu/Zn-O_V-Ce solid solution (peak γ). The order of peak γ ratio over CuZnCe catalysts is as follows: Ce-CuZn> CuZn-Ce> Ce-CuZn-IM.

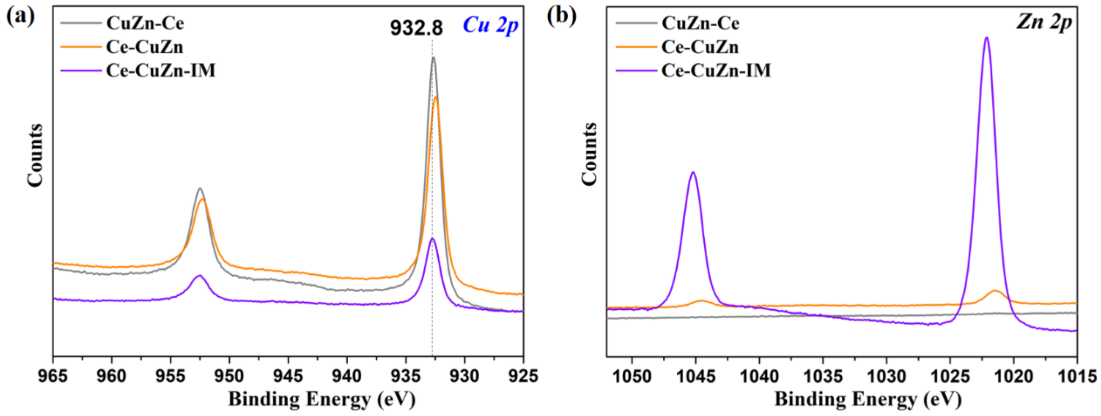

Supplementary Fig. 12. Quasi in-situ XPS profiles of the reduced CuZnCe catalysts: (a) Cu *2p*, (b) Zn *2p*.

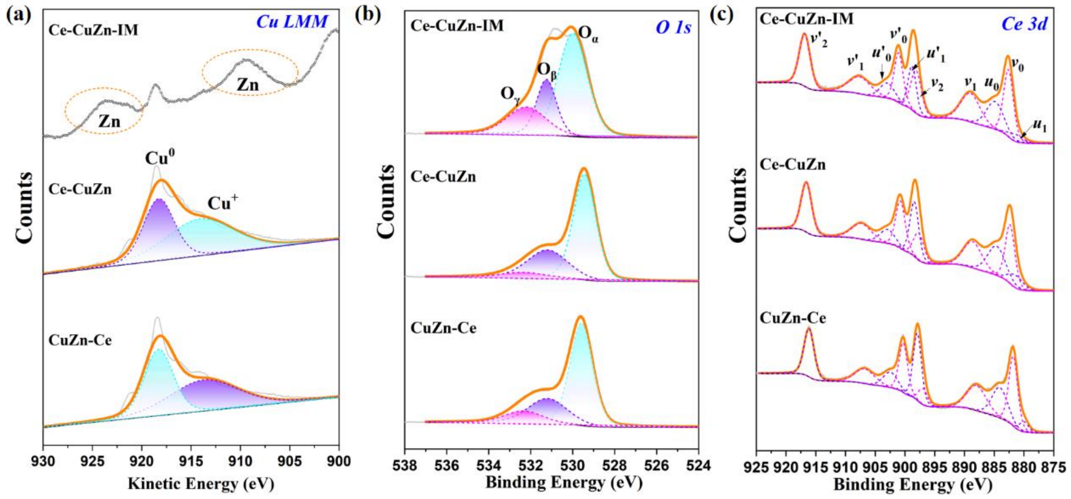

Supplementary Fig. 13. Quasi in-situ XPS profiles of the reduced CuZnCe catalysts: (a) Cu *LMM*, (b) O *1s*, (c) Ce *3d*.

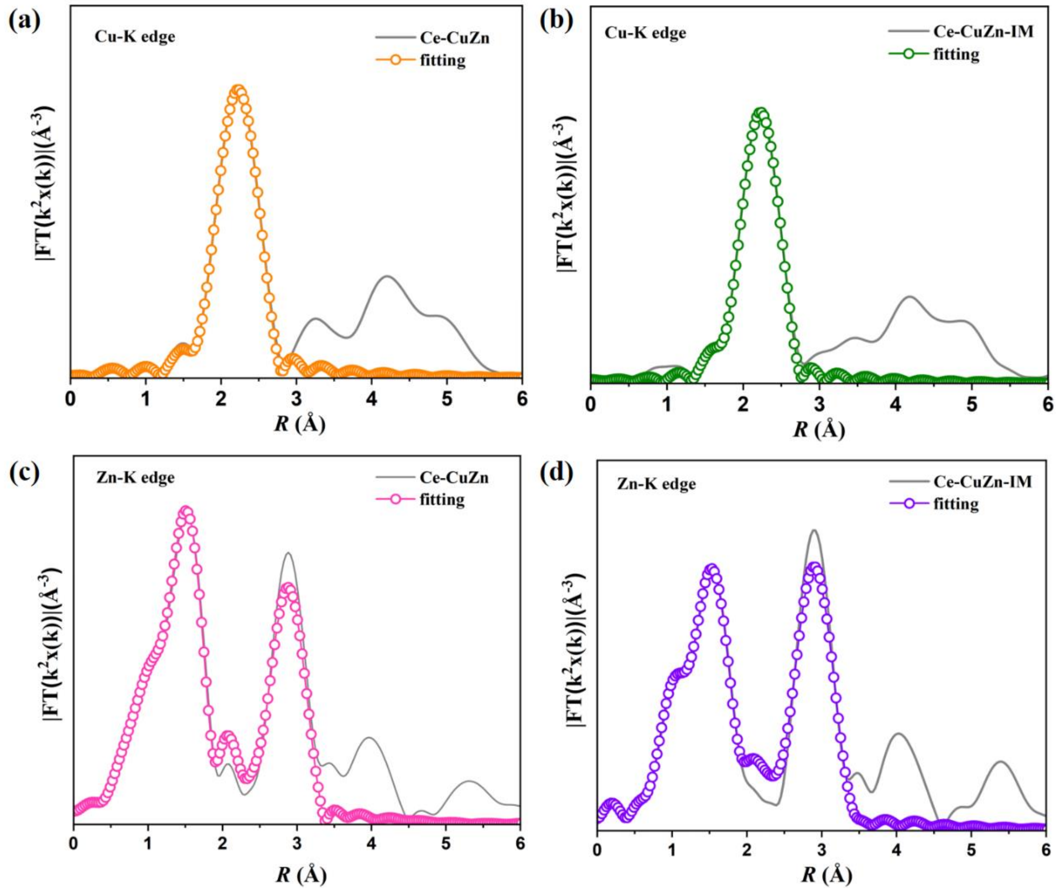

Supplementary Fig. 14. XAS spectra of reduced Ce-CuZn and Ce-CuZn-IM samples: R space Cu K-edge EXAFS spectra and fitting curves for Ce-CuZn (a) and Ce-CuZn-IM (b) samples. *R* space Zn K-edge EXAFS spectra and fitting curves for Ce-CuZn (c) and Ce-CuZn-IM (d) samples.

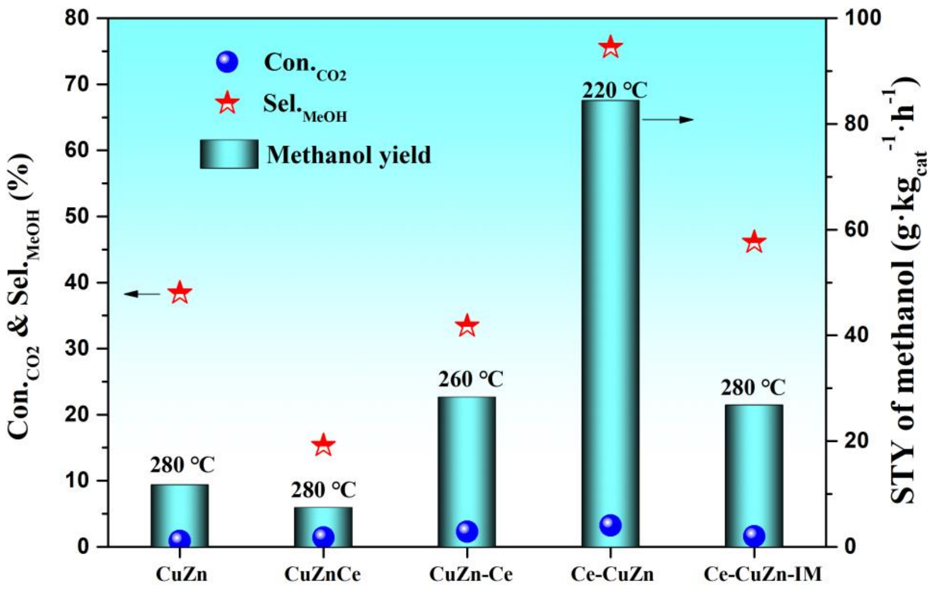

Supplementary Fig. 15. The methanol selectivity and STY of methanol under similar CO_2 conversion. Reaction conditions: *P*= 2.0 MPa, *GHSV*= 10,000 mL·g_{cat}^{-1}·h^{-1}, H_2: CO_2: N_2= 72: 24: 1.

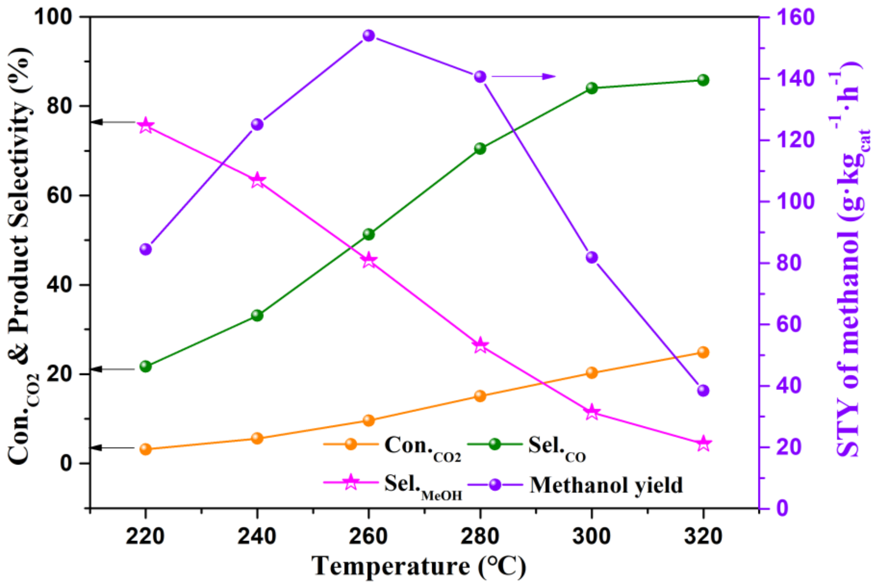

Supplementary Fig. 16. The Con.$_{CO2}$ and methanol selectivity as well as the STY of methanol over the Ce-CuZn catalyst *via* different reaction temperatures in CO_2 hydrogenation. Other reaction conditions: *P*= 2.0 MPa, *GHSV*= 10,000 mL·g_{cat}^{-1}·h^{-1}, H_2: CO_2: N_2= 72: 24: 1.

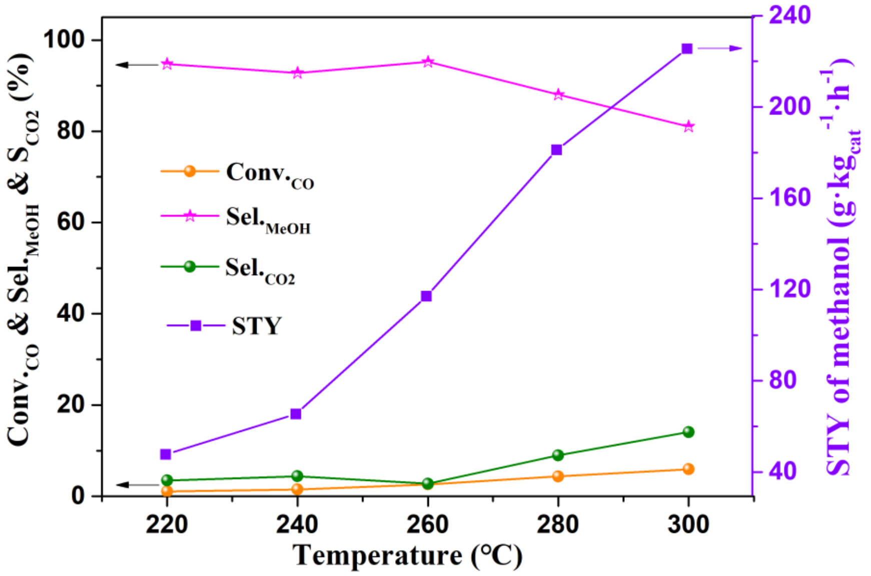

Supplementary Fig. 17. The Con.$_{CO}$ and product selectivity as well as the STY of methanol over the Ce-CuZn catalyst via different reaction temperatures in CO hydrogenation. Other reaction conditions: P= 2.0 MPa, $GHSV$= 10,000 mL·g_{cat}^{-1}·h^{-1}, H_2: CO: N_2= 72: 24: 1. The CO hydrogenation test was similar to that of CO_2 hydrogenation except that the raw reaction gases (H_2: CO: N_2= 72: 24: 1) was changed.

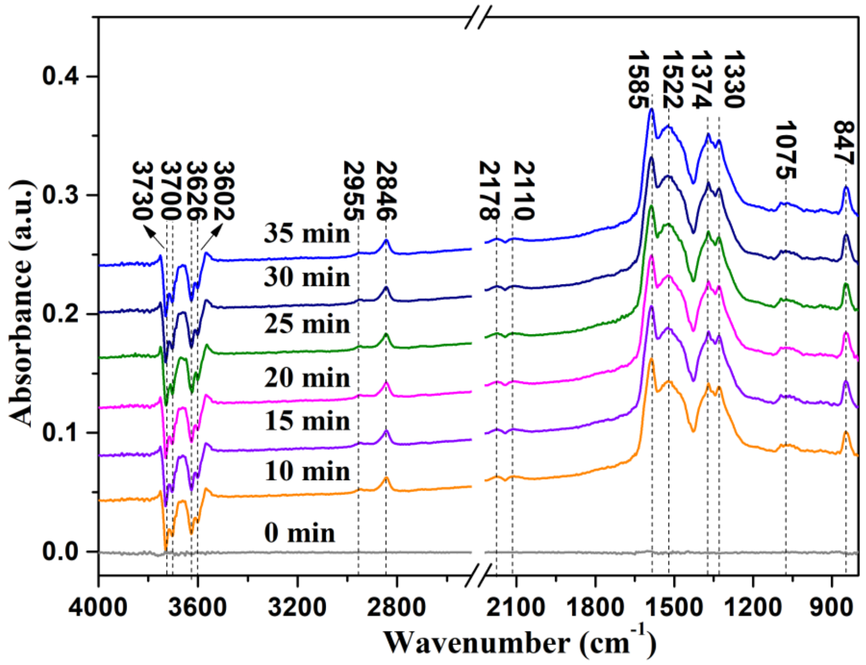

Supplementary Fig. 18. The result of in-situ DRIFTS for CO_2 hydrogenation (10%CO_2-40%H_2/Ar) at 260 °C and 0.1 MPa on Ce-CuZn-IM catalyst. The mains adsorption peaks over Ce-CuZn-IM and Ce-CuZn catalysts are similar except the peaks at around 3730~3602 cm^{-1}, which were attributed to the terminal, bridged, and triply bridged hydroxyls as well as the Ce^{3+}-OH (Fig. 5b)[43]. It means that the hydroxyls groups over Ce-CuZn and Ce-CuZn-IM catalysts were different. The Ce-CuZn-IM catalyst may have already some-OH containing molecules adsorbed at ambient conditions, then set that spectrum as baseline, and under the reaction conditions these molecules desorbed so it was left with negative spectra in this region.

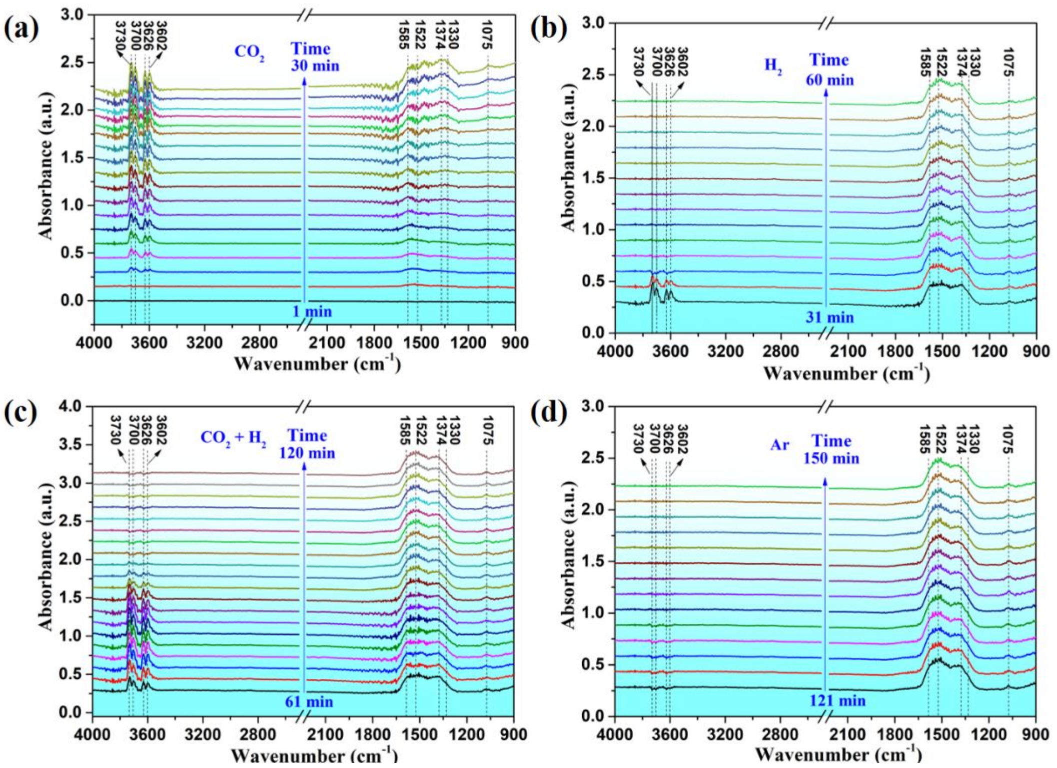

Supplementary Fig. 19. Transient in-situ DRIFTS experiments on Ce-CuZn catalyst collected at 260 °C when the reaction atmospheres were switched from (a) CO_2 to (b) H_2, (c) CO_2+H_2, and (d) Ar. The detailed description of the figure results is shown in the main text, which further demonstrates the formate as an important intermediate.

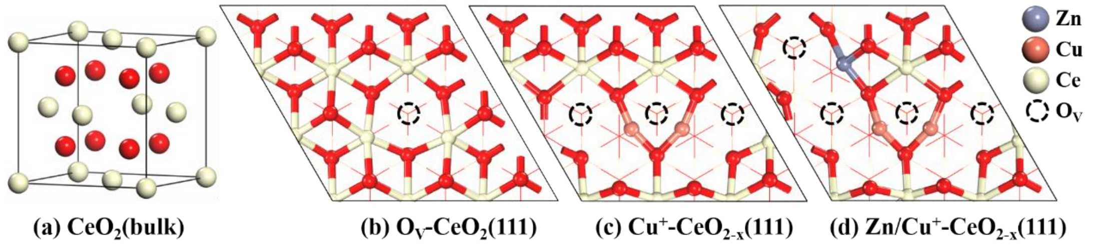

Supplementary Fig. 20. Surface morphology of samples in this work: (a) CeO_2(bulk), (b) O_V-CeO_2(111), (c) Cu^+-CeO_{2-x}(111) and (d) Zn/Cu^+-CeO_{2-x}(111) surfaces. Yellow: Ce, purple: Zn, brown: Cu, red: O, Black dashed circle: oxygen vacancies.

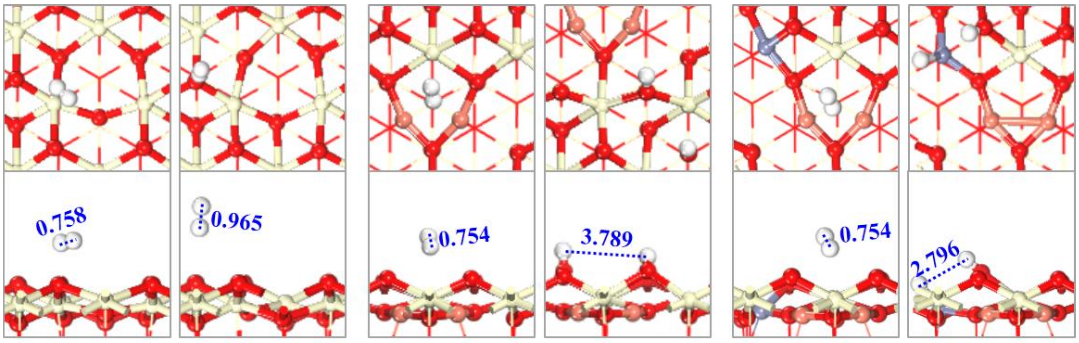

Supplementary Fig. 21. The optimized structures of H_2 adsorption configurations on the models: (a) O_V-CeO_2, (b) Cu^+-CeO_{2-x}, (c) Zn/Cu^+-CeO_{2-x} catalysts. Yellow: Ce, purple: Zn, brown: Cu, red: O, white: H. The specific description of this figure is shown below.

Over O_V-CeO_2, H_2 prefers to be adsorbed at the top-Ce site with the adsorption energy of -19.3 kJ·mol^{-1}, and the H–H bond length is 0.758 Å, which is very close to that in the gas phase (0.751 Å). However, H_2 initially adsorbed at the top-O site becomes spontaneously dissociative to form two H atoms with the slightly stretched H–H distance of 0.965 Å.

Over Cu^+-CeO_{2-x}, H_2 is physisorbed at the Cu-Cu bridge site with the adsorption energy of 23.0 kJ·mol^{-1}, and the H–H bond length is 0.754 Å. However, H_2 initially adsorbed at the top-O site becomes spontaneous dissociation into two H atoms with a largely stretched H–H distance of 3.789 Å, and the dissociated H atoms bonds with the O site to form two surface hydroxyl groups (OH*). Thus, Cu^+ site is weak for molecular adsorption hydrogen, but the adjacent oxygen site promotes H_2 dissociative adsorption.

Over Zn/Cu^+-CeO_{2-x}, H_2 is physisorbed at the Cu-Cu bridge site with the adsorption energy of 1.7 kJ·mol^{-1}, and the H–H bond length is 0.754 Å. Meanwhile, H_2 initially adsorbed at the Zn-O bridge site becomes spontaneous dissociation with the largely stretched H–H distance of 2.796 Å, generating a hydride (M–H) that binds with Zn site and a hydroxyl group (OH*) that bonds with the O site. Herein, it is found that the heterolytic dissociation of H_2 take place at the Zn-O_V site that is ascribed to the polarization of Zn–O bonds, while Cu^+-CeO_{2-x} surface might favor the homolytic dissociation of H_2.

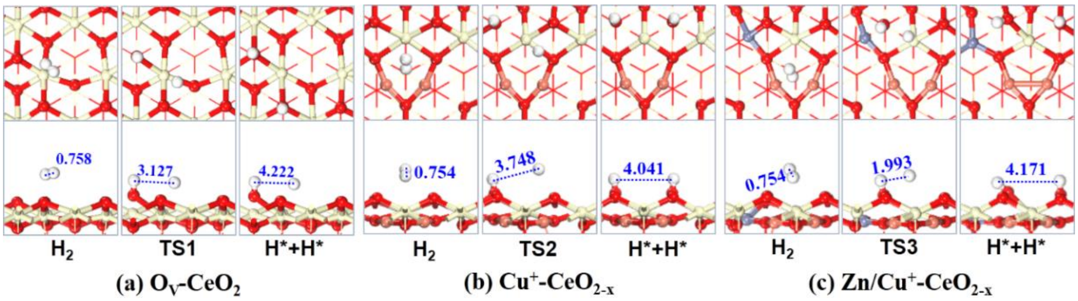

Supplementary Fig. 22. The structures of initial state, transition state and final state involved in the dissociation of molecular adsorption H_2 on the models: (a) O_V-CeO_2, (b) Cu^+-CeO_{2-x}, (c) Zn/Cu^+-CeO_{2-x} catalysts. The color codes are the same as supplementary Fig. 21.

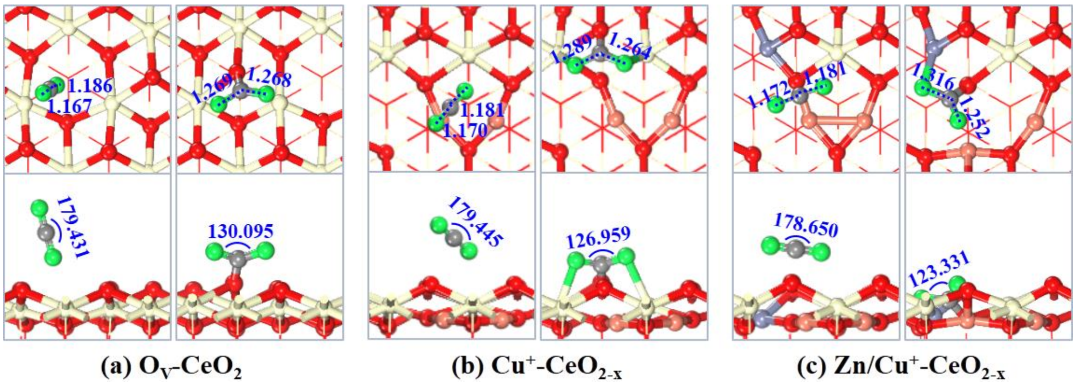

Supplementary Fig. 23. The optimized configurations of both the linear and bent CO_2 adsorption on the models: (a) O_V-CeO_2, (b) Cu^+-CeO_{2-x}, (c) Zn/Cu^+-CeO_{2-x} catalysts, the corresponding adsorption energies and key geometry parameters (the distances of $d_{C\text{–}O}$) are listed in Supplementary Table 7. Yellow: Ce, purple: Zn, brown: Cu, grey: C, red represents the surface O and green represents O in adsorbed molecules.

Over O_V-CeO_2, CO_2 is adsorbed in the linear and bent forms with the adsorption energies of -14.3 and -30.6 kJ·mol^{-1}, respectively. In the linear form, CO_2 is adsorbed at the oxygen vacancy with the C–O bond lengths of 1.167 and 1.186 Å and the O–C–O angle of 179.431°, which is close to that of 1.177 Å and 180° in gas-phase CO_2. In the bent form, CO_2 is adsorbed at the Ce-O bridge site in the form of carbonate, both C

and O atoms of CO_2 are associated with a surface O and Ce atoms, respectively, the C–O bonds are elongated to 1.269 and 1.268 Å, and the O–C–O angle is bent to 130.095°.

Over Cu^+-CeO_{2-x}, CO_2 is also adsorbed in the linear and bent forms with the adsorption energies of 55.9 and -48.2 kJ·mol^{-1}, respectively. In the linear form, CO_2 is adsorbed at the oxygen vacancy near the Cu^+ site. In the bent form, CO_2 is adsorbed at the Ce-O bridge site in the form of carbonate, the optimized CO_2 is bent with the C–O bond lengths (C–O_1 and C–O_2) of 1.289 and 1.264 Å and the O–C–O angle of 126.959°.

Over Zn/Cu^+-CeO_{2-x}, CO_2 is adsorbed in the linear and bent forms with the adsorption energies of 27.1 and -58.5 kJ·mol^{-1}, respectively. In the linear form, CO_2 is adsorbed at the oxygen vacancy near the Cu^+ site. In the bent form, CO_2 adsorbed at the Zn-O bridge site in the form of carbonate, the optimized CO_2 is bent with the C–O bond lengths (C–O_1 and C–O_2) of 1.316 and 1.252 Å and the O–C–O angle of 123.331°.

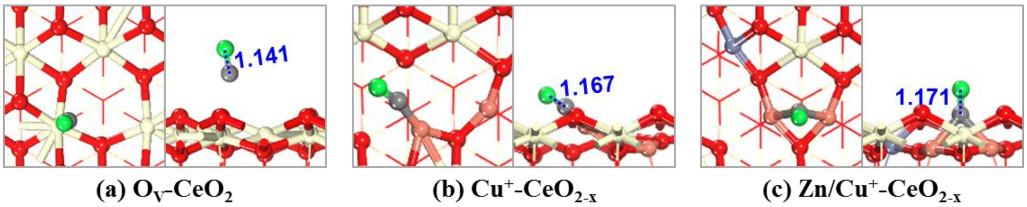

Supplementary Fig. 24. The optimized configurations of CO adsorption on the models: (a) O_V-CeO_2, (b) Cu^+-CeO_{2-x}, (c) Zn/Cu^+-CeO_{2-x} catalysts, the corresponding adsorption energies and key geometry parameters (the distances of d_{C-O}) are listed in Supplementary Table 7. The color codes are the same as supplementary Fig. 23.

Over O_V-CeO_2, CO can be adsorbed at the top of oxygen vacancy with the adsorption energy of -36.7 kJ·mol^{-1} at 0 K, the C−O bond length is 1.141 Å. The calculated result is consistent with the previous studies[43,44], which presents that the CO adsorption energy on the O_V-CeO_2(111) is -34.7 kJ·mol^{-1} at 0 K, and the C−O bond length is 1.150 Å. Over Cu^+-CeO_{2-x}, CO is adsorbed at the Cu^+ top site with the adsorption energy of -61.3 kJ·mol^{-1} at 0 K, and the C−O bond is 1.167 Å. Similarly, Wu et al. showed that CO molecule can be strongly bonded to Cu^+ species on the Cu^{m+}-CeO_x catalyst with the adsorption energy of -55.96 kJ·mol^{-1} at 0 K [45]. Over Zn/Cu^+-

CeO_{2-x}, CO is adsorbed at the Cu-Cu bridge site with the adsorption energy of -87.5 kJ·mol^{-1} at 0 K, and the C−O bond length is 1.171 Å.

Thus, the adsorption and activation ability of CO over O_V-CeO_2, Cu^+-CeO_{2-x}, and Zn/Cu^+-CeO_{2-x} catalysts follow the order of Zn/Cu^+-CeO_{2-x} >Cu^+-CeO_{2-x}>O_V-CeO_2. Moreover, the most favorable adsorption site of CO is Cu^+ species both on the Cu^+-CeO_{2-x} and Zn/Cu^+-CeO_{2-x} catalysts.

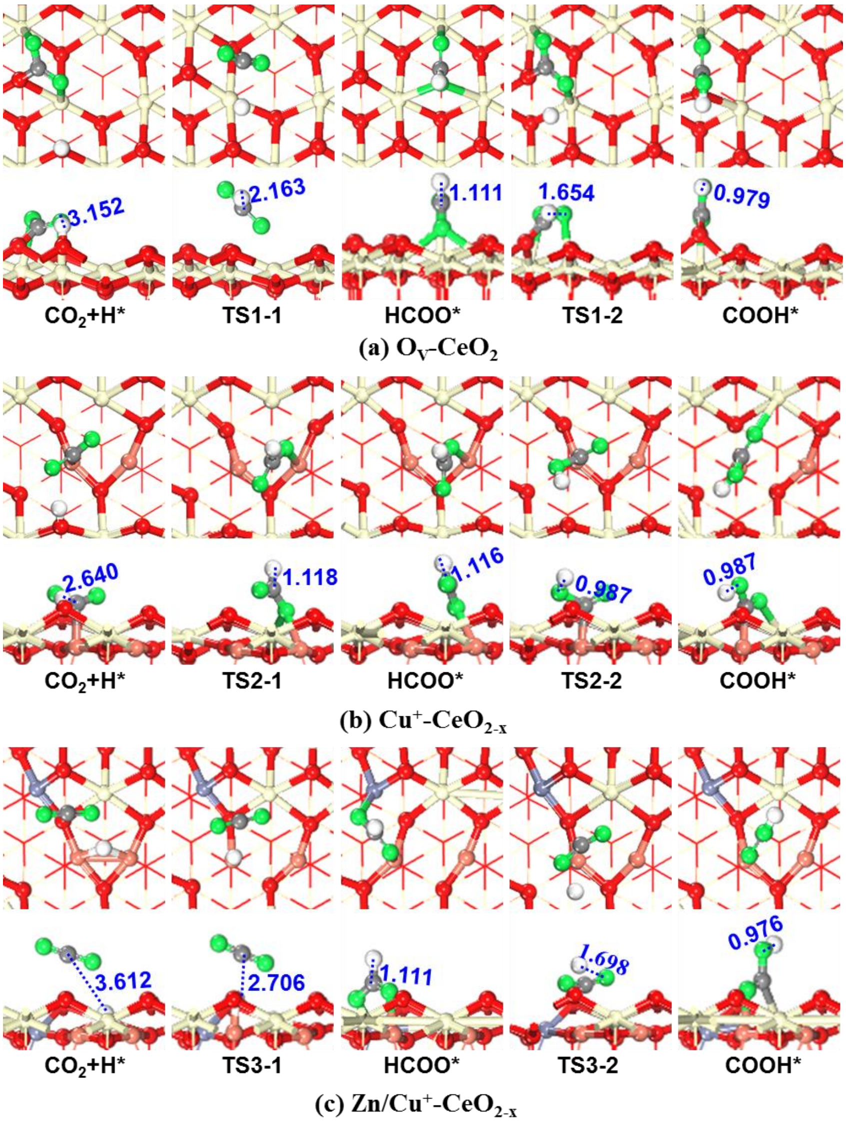

Supplementary Fig. 25. The structures of initial state, transition state and final state of CO_2 activation on the models: (a) O_V-CeO_2, (b) Cu^+-CeO_{2-x}, (c) Zn/Cu^+-CeO_{2-x} catalysts. The color codes are the same as supplementary Fig. 23.

Supplementary References

1. Wang, J. *et al.* A highly selective and stable ZnO-ZrO_2 solid solution catalyst for CO_2 hydrogenation to methanol. *Sci. Adv.* **3**, e1701290 (2017).
2. Ye, R. P. *et al.* Engineering Ni/SiO_2 catalysts for enhanced CO_2 methanation. *Fuel* **285**, 119151 (2021).
3. Ye, R. P. *et al.* Fabrication method engineered Cu-ZnO/SiO_2 catalysts with highly dispersed metal nanoparticles toward efficient utilization of methanol as a hydrogen carrier. *Adv. Energy Sustainability Res.* **2**, 2100082 (2021).
4. Zhu, J. *et al.* Mechanism and nature of active sites for methanol synthesis from CO/CO_2 on Cu/CeO_2. *ACS Catal.* **10**, 11532-11544 (2020).
5. Kresse, G. & Furthmüller, J. Efficient iterative schemes for *ab* initio total-energy calculations using a plane-wave basis set. *Phy. Rev. B* **54**, 11169-11186 (1996).
6. Kresse, G. & Hafner, J. *Ab* initio molecular dynamics for open-shell transition metals. *Phy. Rev. B* **48**, 13115-13118 (1993).
7. Perdew, J. P. *et al.* Atoms, molecules, solids, and surfaces: Applications of the generalized gradient approximation for exchange and correlation. *Phy. Rev. B* **46**, 6671-6687 (1992).
8. Liu, X. *et al.* Activation of subnanometric Pt on Cu-modified CeO_2 via redox-coupled atomic layer deposition for CO oxidation. *Nat. Commun.* **11**, 4240 (2020).
9. Sheppard, D., Xiao, P., Chemelewski, W., Johnson, D. D. & Henkelman, G. A generalized solid-state nudged elastic band method. *J. Chem. Phys.* **136**, 074103 (2012).
10. Liu, X. M., Lu, G. Q. & Yan, Z. F. Nanocrystalline zirconia as catalyst support in methanol synthesis. *Appl. Catal. A: Gen.* **279**, 241-245 (2005).
11. Olsen, R. A., Kroes, G. J., Henkelman, G., Arnaldsson, A. & Jonsson, H. Comparison of methods for finding saddle points without knowledge of the final states. *J. Chem. Phys.* **121**, 9776-9792 (2004).
12. Henkelman, G. & Jónsson, H. A dimer method for finding saddle points on high dimensional potential surfaces using only first derivatives. *J. Chem. Phys.* **111**, 7010-7022 (1999).
13. Wang, V., Xu, N., Liu, J. C., Tang, G. & Geng, W. T. VASPKIT: a pre-and post-processing program for VASP code. *arXiv preprint arXiv* **1908**, 08269 (2019).

14. Lu, X. *et al.* Initial reduction of CO_2 on perfect and O-defective CeO_2 (111) surfaces: towards CO or COOH? *RSC Adv.* **5**, 97528-97535 (2015).

15. Whiteld, H. J., Roman, D. & Palmer, A. R. X-ray study of the system ThO_2-CeO_2-Ce_2O_3. *J. Inorg. Nucl. Chem.* **28**, 2817-2825 (1966).

16. Wang, Z. Q. *et al.* High-performance and long-lived Cu/SiO_2 nanocatalyst for CO_2 hydrogenation. *ACS Catal.* **5**, 4255-4259 (2015).

17. Yang, H. Y. *et al.* Core–shell structured Cu@m-SiO_2 and Cu/ZnO@m-SiO_2 catalysts for methanol synthesis from CO_2 hydrogenation. *Catal. Commun.* **84**, 56-60 (2016).

18. Jiang, Y. *et al.* Slurry methanol synthesis from CO_2 hydrogenation over micro-spherical SiO_2 support Cu/ZnO catalysts. *Journal of CO_2 Utilization* **26**, 642-651 (2018).

19. Shao, Y., Kosari, M., Xi, S. & Zeng, H. C. Single solid precursor-derived three-dimensional nanowire networks of CuZn-silicate for CO_2 hydrogenation to methanol. *ACS Catal.* **12**, 5750-5765 (2022).

20. Toyir, J., de la Piscina, P. R., Fierro, J. L. G. & Homs, N. Highly effective conversion of CO_2 to methanol over supported and promoted copper-based catalysts: influence of support and promoter. *Appl. Catal. B: Environ.* **29**, 207-215 (2001).

21. Gao, P. *et al.* Influence of modifier (Mn, La, Ce, Zr and Y) on the performance of Cu/Zn/Al catalysts via hydrotalcite-like precursors for CO_2 hydrogenation to methanol. *Appl. Catal. A: Gen.* **468**, 442-452 (2013).

22. Lei, H., Hou, Z. & Xie, J. Hydrogenation of CO_2 to CH_3OH over CuO/ZnO/Al_2O_3 catalysts prepared via a solvent-free routine. *Fuel* **164**, 191-198 (2016).

23. Tan, Q., Shi, Z. & Wu, D. CO_2 hydrogenation to methanol over a highly active Cu−Ni/CeO_2-nanotube natalyst. *Ind. Eng. Chem. Res.* **57**, 10148-10158 (2018).

24. Hu, J. *et al.* Sulfur vacancy-rich MoS_2 as a catalyst for the hydrogenation of CO_2 to methanol. *Nat. Catal.* **4**, 242-250 (2021).

25. An, B. *et al.* Confinement of ultrasmall Cu/ZnO_x nanoparticles in metal-organic frameworks for selective methanol synthesis from catalytic hydrogenation of CO_2. *J. Am. Chem. Soc.* **139**, 3834-3840 (2017).

26. Gao, P. *et al.* Yttrium oxide modified Cu/ZnO/Al_2O_3 catalysts via hydrotalcite-like precursors for CO_2 hydrogenation to methanol. *Catal. Sci. Technol.* **5**, 4365-4377 (2015).

27. Wang, X. *et al.* Catalytic activity for direct CO_2 hydrogenation to dimethyl ether with different proximity of bifunctional Cu-ZnO-Al_2O_3 and ferrierite. *Appl. Catal. B: Environ.* **327**, 122456 (2023).

28. Chen, K. *et al.* CO_2 hydrogenation to methanol over Cu catalysts supported on La-modified SBA-15: The crucial role of Cu–LaO_x interfaces. *Appl. Catal. B: Environ.* **251**, 119-129 (2019).

29. Guo, X., Mao, D., Lu, G., Wang, S. & Wu, G. CO_2 hydrogenation to methanol over Cu/ZnO/ZrO_2 catalysts prepared via a route of solid-state reaction. *Catal. Commun.* **12**, 1095-1098 (2011).

30. Wu, C. *et al.* Inverse ZrO_2/Cu as a highly efficient methanol synthesis catalyst from CO_2 hydrogenation. *Nat. Commun.* **11**, 5767 (2020).

31. Wang, W., Qu, Z., Song, L. & Fu, Q. CO_2 hydrogenation to methanol over Cu/CeO_2 and Cu/ZrO_2 catalysts: Tuning methanol selectivity via metal-support interaction. *J. Energy Chem.* **40**, 22-30 (2020).

32. Zhang, C. *et al.* Oxygen vacancies in Cu/TiO_2 boost strong metal-support interaction and CO_2 hydrogenation to methanol. *J. Catal.* **413**, 284-296 (2022).

33. Zhu, J. *et al.* Flame synthesis of Cu/ZnO-CeO_2 catalysts: Synergistic metal-support interactions promote CH_3OH selectivity in CO_2 hydrogenation. *ACS Catal.* **11**, 4880-4892 (2021).

34. Choi, E. J., Lee, Y. H., Lee, D.-W., Moon, D.-J. & Lee, K.-Y. Hydrogenation of CO_2 to methanol over Pd–Cu/CeO_2 catalysts. *Mol. Catal.* **434**, 146-153 (2017).

35. Tan, Q., Shi, Z. & Wu, D. CO_2 hydrogenation over differently morphological CeO_2 supported Cu-Ni catalysts. *Int. J. Energy Res.* **43**, 5392-5404 (2019).

36. Li, S., Guo, L. & Ishihara, T. Hydrogenation of CO_2 to methanol over Cu/AlCeO catalyst. *Catal. Today* **339**, 352-361 (2020).

37. Zhang, C., Liao, P., Wang, H., Sun, J. & Gao, P. Preparation of novel bimetallic CuZn-BTC coordination polymer nanorod for methanol synthesis from CO_2 hydrogenation. *Mater. Chem. Phys.* **215**, 211-220 (2018).

38. Sun, K. H. *et al.* Hydrogenation of CO_2 to methanol over In_2O_3 catalyst. *J. CO_2 Util.* **12**, 1-6 (2015).

39. Dang, S. *et al.* Rationally designed indium oxide catalysts for CO_2 hydrogenation to methanol with high activity and selectivity. *Sci. Adv.* **6**, eaaz2060 (2020).

40. Pustovarenko, A. *et al.* Metal-organic framework-derived synthesis of cobalt indium catalysts for the hydrogenation of CO_2 to methanol. *ACS Catal.* **10**, 5064-5076 (2020).

41. Li, X., Liu, G., Xu, D., Hong, X. & Tsang, S. C. E. Confinement of subnanometric PdZn at a defect enriched ZnO/ZIF-8 interface for efficient and selective CO_2 hydrogenation to methanol. *J. Mater. Chem. A* **7**, 23878–23885 (2019).

42. Rasteiro, L. F. *et al.* Insights into the alloy-support synergistic effects for the CO_2 hydrogenation towards methanol on oxide-supported Ni_5Ga_3 catalysts: An experimental and DFT study. *Appl. Catal. B: Environ.* **302**, 120842 (2022).

43. Guo, Y. *et al.* Low-temperature CO_2 methanation over CeO_2-supported Ru single atoms, nanoclusters, and nanoparticles competitively tuned by strong metal–support interactions and H-spillover effect. *ACS Catal.* **8**, 6203-6215 (2018).

44. Jiang, S. Y., Teng, B. T., Yuan, J. H. G., X. W. & Luo, M. F. Adsorption and Oxidation of CO over CeO_2(111) Surface. *Acta Phys.-Chim. Sin.* **25**, 1629-1634 (2009).

45. Wu, D. *et al.* Cuprous ions embedded in ceria lattice for selective and stable electrochemical reduction of carbon dioxide to ethylene. *J. Mater. Chem. A* **6**, 9373-9377 (2018).
